# ﻿Genera *Colastes* Haliday, *Colastinus* Belokobylskij, and *Xenarcha* Foerster (Hymenoptera, Braconidae, Exothecinae) from the Korean Peninsula with a discussion on the Exothecinae genus and subgenus composition

**DOI:** 10.3897/zookeys.1236.148928

**Published:** 2025-04-29

**Authors:** Sergey A. Belokobylskij, Deokseo Ku

**Affiliations:** 1 Zoological Institute of the Russian Academy of Sciences, St. Petersburg 199034, Russia Zoological Institute of the Russian Academy of Sciences St. Petersburg Russia; 2 The Science Museum of Natural Enemies, Geochang 50147, Republic of Korea The Science Museum of Natural Enemies Geochang Republic of Korea

**Keywords:** Asia, descriptions, Ichneumonoidea, keys, *Pseudophanomeris*, parasitoid, *Shawiana*

## Abstract

The Exothecinae genera *Colastes* Haliday, 1833, *Colastinus* Belokobylskij, 1984, and *Xenarcha* Foerster, 1863 of the Korean peninsula are reviewed. The names *Pseudophanomeris* Belokobylskij, 1984 and *Shawiana* van Achterberg, 1983 are synonymised with the genus *Xenarcha* Foerster and treated as subgenera. The two new species of *Colastes* and one new species and subspecies of *Xenarcha* are described and illustrated. *Exothecuseffectus* Papp, 1972 is included in *Xenarcha* Foerster, **comb. nov.** The composition and distribution of the world-known Exothecinae genera are discussed and an illustrated key to its genera and subgenera is presented. A key to the Korean species of the genera *Colastes*, *Xenarcha*, and *Colastinus* is also provided.

## ﻿Introduction

The subfamily Exothecinae is one of the small taxonomical groups of the cyclostome phylogenetic clade of the family Braconidae with reduced number of the genera in the World fauna. Previous to the current publication, seven genera were included in this subfamily, namely *Colastes* Haliday, 1833, *Colastinus* Belokobylskij, 1984, *Hormiopius* Blanchard, 1962, *Orientocolastes* Belokobylskij, 1999, *Shawiana* van Achterberg, 1983, *Vietcolastes* Belokobylskij, 1992, and *Xenarcha* Foerster, 1863 (Yu et al. 2016). However, the name *Hormiopius* Blanchard was recently synonymized under *Heterospilus* Haliday, 1836, a member of subfamily Doryctinae (Martínez and Diez 2024). The status of *Shawiana* and *Xenarcha* has also changed and they have been treated only as subgenera of *Colastes* (Belokobylskij 1998).

The Palaearctic fauna of this subfamily includes only three genera *Colastes*, *Colastinus* and *Xenarcha*. *Shawiana* and *Pseudophanomeris* Belokobylskij, 1984 are treated here only as subgenera of *Xenarcha* (syn. nov.). On the other hand, the species diversity of *Colastes* and *Xenarcha* in this realm is relatively large and includes 23 and 28 already known species respectively. The information on the species of subfamily Exothecinae from the Korean peninsula is sparse and scattered (Papp 1987, 1992, 2003; Belokobylskij 1998; Ku et al. 2001).

In this paper we include illustrated descriptions of the two new species of *Colastes*, and the new species and subspecies of *Xenarcha*, a key to all species recorded on the Korean peninsula, and the original key to all known Exothecinae genera and subgenera.

## ﻿Materials and methods

The terminology employed for the morphological features, sculpture, and body measurements follows Belokobylskij and Maetô (2009) and Belokobylskij et al. (2024). The wing venation nomenclature follows Belokobylskij and Maetô (2009) and Belokobylskij et al. (2024), with the terminology of van Achterberg (1993) shown in parentheses. The new distribution records presented in this paper are marked with an asterisk (*). In the key, additional features useful for separating taxa are listed after an dash (–).The specimens were examined using an Olympus SZ51 microscope. Photographs were taken with an Olympus OM-D E-M1 digital camera mounted on an Olympus SZX10 microscope (Zoological Institute of the Russian Academy of Sciences, St Petersburg, Russia). Image stacking was performed using Helicon Focus 8.0. The figures were produced using Adobe Photoshop CS6.The specimens examined in this study are deposited in the collections of the National Institute of Biological Resources (Incheon, Republic of Korea; **NIBR**), the Science Museum of Natural Enemies (Geochang, Republic of Korea; **SMNE**), and the Zoological Institute of the Russian Academy of Sciences (St Petersburg, Russia; **ZISP**).Abbreviations of Korean provinces used in this paper as follows: **CB** – Chungcheongbuk-do, **CN** – Chungcheongnam-do, **GB** – Gyeongsangbuk-do, **GG** – Gyeonggi-do, **GN** – Gyeongsangnam-do, **GW** – Gangwon-do, **JB** – Jeollabuk-do, **JJ** - Jeju-do, **JN** – Jeollanam-do.

## ﻿Taxonomy

### ﻿Class Insecta Linnaeus, 1758

**Order Hymenoptera Linnaeus, 1758**

**Family Braconidae Nees, 1811**

**Subfamily Exothecinae Foerster, 1863**

#### ﻿Genus Colastes

Taxon classificationAnimaliaHymenopteraBraconidae

Haliday, 1833

2C070E32-841E-506A-B589-F08372ACFECA

##### Type species.

*Colastesbraconius* Haliday, 1833.

#### ﻿ Colastes (Colastes) braconius

Taxon classificationAnimaliaHymenopteraBraconidae

Haliday, 1833

E9E78F37-7D9D-5364-9865-93A40A9BF749

Fig. 13

Colastes braconius Haliday, 1833: 266; Shenefelt 1975: 1117; Belokobylskij and Tobias 1986: 57; Belokobylskij 1998: 143; Ku et al. 2001: 153; Yu et al. 2016; Belokobylskij et al. 2019: 281.
Exothecus debilis Wesmael, 1838: 75; Shenefelt 1975: 1118.
Colastes gracilis Papp, 1975: 411; Belokobylskij 1994: 69 (as synonym); 1998: 143.

##### Material examined.

South Korea: [GW] • Goseong-gun, Ganseong-eup, Jinbu-ri, Hyangrobong-peak (DMZ), 13.VI.1992 (J.-W. Lee), 4 females, 1 male (SMNE, ZISP) • Hyangrobong, Gangwondo, 13.VI.1992 (J.-W. Lee), 1 female (SMNE).

##### Hosts.

Polyphagous species, its hosts include the larval stages of flies (Diptera) of the family Agromyzidae, moths (Lepidoptera) of the families Coleophoridae, Cosmopterigidae, Elachistidae, Eriocraniidae, Gracillariidae, Heliozelidae, Lycaenidae, Lyonetiidae, Momphidae, Nepticulidae, Pyralidae, Tischeriidae, Tortricidae, and Ypsolophidae, beetles (Coleoptera) of the family Curculionidae, and sawflies (Hymenoptera) of the family Tenthredinidae (Yu et al. 2016; Belokobylskij et al. 2019).

##### Distribution.

Korean peninsula; Tunisia, Europe (widely), Caucasus, Turkey, Iran, Kazakhstan, Russia (European part, Urals, Siberia, Far East), Japan.

#### ﻿ Colastes (Colastes) dersu

Taxon classificationAnimaliaHymenopteraBraconidae

Belokobylskij, 1998

EFA8EF46-9F04-5991-A5B6-6668670CCADE

Colastes (Colastes) dersu Belokobylskij, 1998: 142; Ku et al. 2001: 155; Yu et al. 2016; Belokobylskij et al. 2019: 281.

##### Material examined.

South Korea [GN] • Hamyang-gun, Macheon-myeon, Samjeong-Byeoksoryeong, Mt. Jiri, 29.VII.1992 (Deokseo Ku), 1 male (SMNE) • Changyeong-gun, Yueo-myeon, Daedae-ri, Upo-swamp, 3.VII.2015 (E. Tselikh), 1 female (ZISP).

[GW] • Goseong-gun, Ganseong-eup, Jinbu-ri, Hyangrobong-peak (DMZ), 13.VI.1992 (J.-W. Lee), 1 female (SMNE) • Yanggu-gun, Dong-myeon, Panlrang-ri, Mt. Daeamsan (DMZ), 30.V.1992 (collector unknown), 1 female (SMNE) • Taebaek-shi, Mt. Taebaek, 13.VIII.1989 (S.M. Ryu), 1 female, 1 male (paratypes) (SMNE).

##### Host.

Unknown.

##### Distribution.

Korean Peninsula; Russia (southern Far East).

#### ﻿ Colastes (Colastes) fragiloides

Taxon classificationAnimaliaHymenopteraBraconidae

Belokobylskij & Ku sp. nov.

193B5D17-E2AC-5EE1-A032-9E005587E3B3

https://zoobank.org/B8A7CB58-FCCF-49C8-9641-DAC0D711CF42

Figs 1 , 2

##### Type material.

***Holotype*** • female, “Korea (GN) Namhae-gun, Seo-myeon, Nogu-ri, Temple Mangunsa 15.X–14.XI.2022 (Malaise trap), Deokseo Ku, Jaehyeon Lee, Hyojin Jeong,” (NIBR). ***Paratypes*** • 3 females, with same label as in holotype (SMNE, ZISP).

##### Description.

**Female**. ***Body*** length 2.1–2.5 mm; fore wing length 2.2–2.8 mm. ***Head*** width 1.7–1.8× its medial length (dorsal view), 1.2× wider than mesoscutum. Temple behind eye weakly convex in anterior 1/2 and distinctly curvedly narrowed in posterior 1/2. Transverse diameter of eye 1.1–1.3× longer than temple (dorsal view). Ocelli small, arranged almost in equilateral triangle. POL approximately equal to Od, 0.3–0.4× OOL. Eye oval, glabrous, 1.25–l.30× as high as broad (lateral view). Malar space ~ 0.4× height of eye, 1.0–1.2× basal width of mandible. Malar suture indistinct or very shallow. Face width 1.1–1.2× height of face and clypeus combined, almost equal to height of eye. Hypoclypeal depression suboval, its width 1.0–1.1× distance from edge of depression to eye, ~ 0.4× width of face. Head distinctly and almost linearly narrowed below eyes. ***Antenna*** rather slender, filiform, 28–29-segmented, 1.2–1.3× longer than body. First flagellar segment 4.0–4.5× longer than its apical width, 1.05–1.15× longer than second segment. Penultimate segment 2.8–3.0× longer than wide, ~ 0.6× as long as first segment, 0.9–1.0× as long as apical segment; the latter acuminate apically and without spine.

***Mesosoma*** 1.9–2.0× longer than its height. Pronotum dorsally with wide, lens-shaped shallow sculptured transverse groove, with distinct and curved medially transverse carina. Mesoscutum distinctly and curvedly elevated above prothorax. Notauli distinct in anterior 0.6, shallow to almost partly absent in posterior 0.4, entirely smooth. Prescutellar depression distinctly rugulose-crenulate, 0.3–0.4× as long as scutellum. Scutellum without transverse furrow and smooth posteriorly. Precoxal sulcus absent. ***Wings.*** Fore wing 2.6–2.8× longer than its maximum width. Pterostigma narrow, 6.0–7.0× its maximum width, 0.8–0.9× as long as metacarp (1-R1). Radial vein (r) arising before middle of pterostigma, from its basal 0.3. Second radial abscissa (3-SR) 2.0–2.7× longer than first abscissa (r) and forming obtuse angle with it, 0.45–0.50× as long as the straight third abscissa (SR1), 1.3–1.4× longer than first radiomedial vein (2-SR). Recurrent vein (m-cu) weakly antefurcal. Second radiomedial (submarginal) cell weakly or very weakly narrowed distally, its length 2.4–2.6× maximum width, 1.7–1.8× longer than brachial (subdiscal) cell. First abscissa of medial vein (1-SR+M) weakly sinuate. Distance (1-CU1) from nervulus (cu-a) to basal vein (1-M) 2.0–2.3× nervulus (cu-a) length. Parallel vein (CU1a) arising from posterior 0.3 of distal margin (3-CU1) of brachial (subdiscal) cell. In hind wing, first abscissa of mediocubital vein (M+CU) ~ 0.8× as long as second abscissa (1-M). First abscissa of costal vein (C+SC+R) 0.5× as long as second abscissa (1-SC+R). Recurrent vein (m-cu) short or very short, sclerotised, weakly antefurcal. ***Legs***. Hind femur 5.2–5.5× longer than wide. Inner spur of hind tibia ~ 0.2× as long as hind basitarsus. Hind tarsus as long as hind tibia. Hind basitarsus 0.6× as long as combined length of second to fifth segments. Second tarsal segment of hind leg 0.5× as long as basitarsus, 1.5–1.7× as long as fifth segment (without pretarsus).

**Figure 1.**
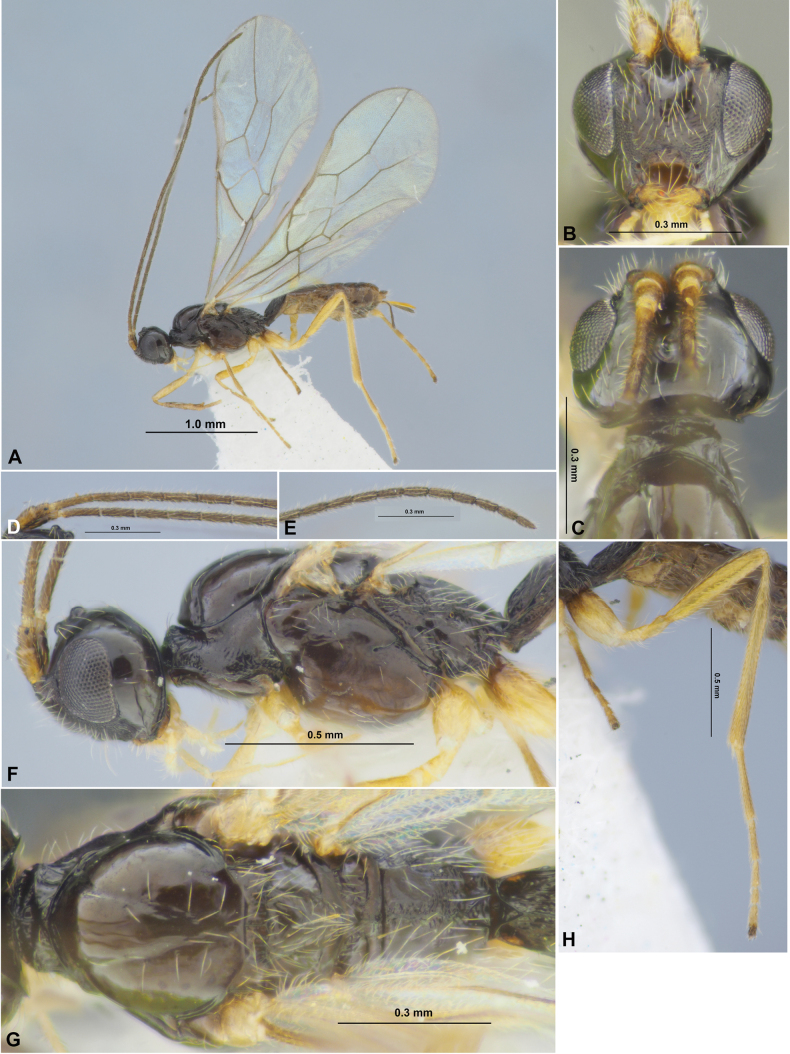
Colastes (Colastes) fragiloides sp. nov., female, holotype **A** habitus, lateral view **B** head, front view **C** head and anterior part of mesosoma, dorsal view **D** basal segments of antenna **E** apical segments of antenna **F** head and mesosoma, lateral view **G** mesosoma, dorsal view **H** hind leg.

***Metasoma*** as long as head and mesosoma combined. First tergite slender, evenly and linearly widened from base to apex, with weak spiracular tubercles, with dorsal carinae fused in basal quarter. Length of first tergite 1.2–1.3× its anterior width, anterior width 2.0–2.2× its posterior width. Second suture very fine, weakly curved, smooth. Medial length of second tergite 0.80–0.85× its anterior width, 1.2–1.3× length of third tergite. Setose part of ovipositor sheath 0.3–0.4× as long as metasoma, 1.2–1.6× longer than first tergite, 1.1–1.6× hind basitarsus, 0.15–0.20× as long as fore wing.

**Figure 2.**
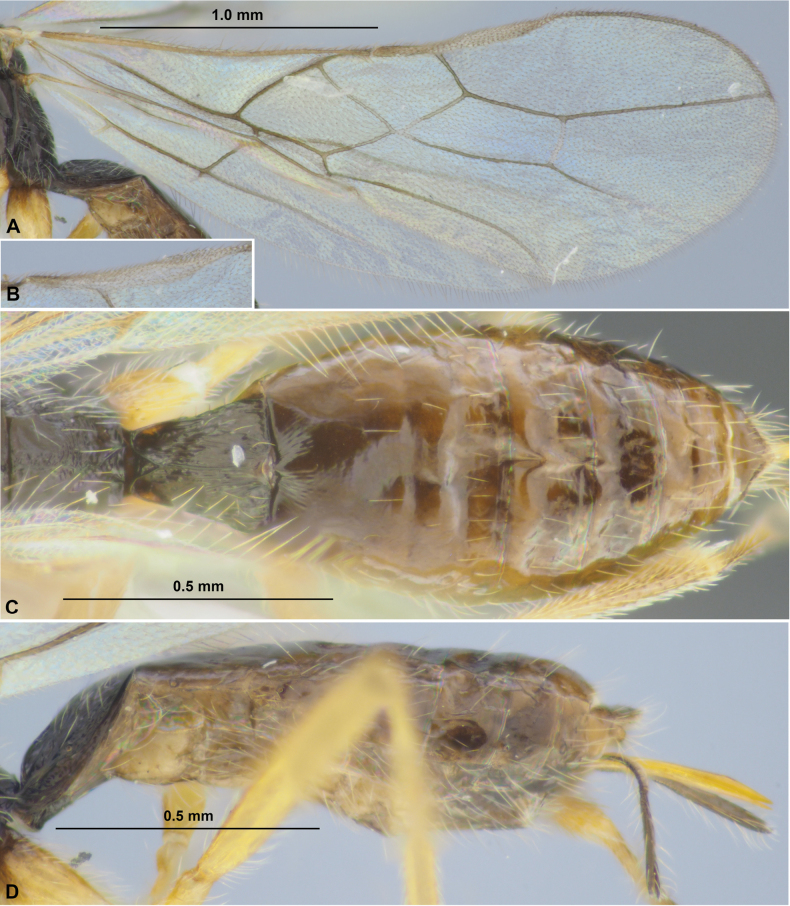
Colastes (Colastes) fragiloides sp. nov., female, holotype **A** wings **B** pterostigma **C** propodeum and metasoma, dorsal view **D** metasoma and ovipositor, lateral view.

***Sculpture and pubescence.*** Vertex and temple smooth; face smooth in upper half and densely reticulate-granulate in lower half or in lower lateral 0.7. Mesoscutum entirely smooth, without medio-posterior sculptured area; scutellum and mesopleuron smooth. Metapleuron almost entirely smooth, weakly rugulose anteriorly and posteriorly. Propodeum with medial carina in anterior 1/2, without delineated areas, smooth to almost smooth in anterior 0.3–0.4, densely reticulate-striate posteriorly. Legs smooth. First metasomal tergite entirely and densely rugose-striate; second tergite mostly or entirely smooth, rarely obliquely striate basally in short area. Remaining tergites smooth. Mesoscutum with sparse, semi-erect and long pale setae along notauli. Hind tibia dorsally with short, dense and semi-erect pale setae, length of these setae ~ 0.5× as long as maximum width of hind tibia.

***Colour.*** Body mainly black; metasoma behind first tergite brown to dark brown. Antennae mainly dark brown to black, four or five basal segments reddish brown. Palpi yellow. Legs mainly yellow, hind coxa basally brown. Wings hyaline. Pterostigma pale yellow.

**Male**. Unknown.

##### Discussion.

This new species is very similar to Colastes (Colastes) fragilis (Haliday, 1836), but differs from later by having the first metasomal tergite longer, 1.2–1.3× longer than its apical width (shorter, not longer than its apical width in *C.fragilis*), the pronotum dorsally with lens-shaped shallow depression (without depression in *C.fragilis*), the propodeum mostly smooth in basal areas and with distinct basal carina (entirely rugose-reticulate or smooth only on small anterior areas and without basal carina in *C.fragilis*), fore wing first abscissa of radial vein (r) long and situated distinctly oblique to pterostigma (short and subvertical or only weakly oblique in *C.fragilis*), upper part of pronotum side and metapleuron mainly smooth (mainly rugose-reticulate in *C.fragilis*), and the second tergite sometimes striate medio-basally (entirely smooth in *C.fragilis*).

##### Etymology.

Named from a combination of the species name *fragilis* and the Latin suffix -*oides* (resembling) because the new species is similar to *Colastes fragilis*.

##### Distribution.

Korean peninsula.

#### ﻿ Colastes (Colastes) interdictus

Taxon classificationAnimaliaHymenopteraBraconidae

Belokobylskij, 1998

5D034FFD-339A-54A9-946E-8E49D796499D

Colastes (Colastes) interdictus Belokobylskij, 1998: 143; Ku et al. 2001: 155; Papp 2003: 118; Yu et al. 2016; Belokobylskij et al. 2019: 281.

##### Material examined.

South Korea: [GB] • Bonghwa-gun, Myeongho-myeon, Gwanchang-ri, Mt. Cheongryang, 14.VII.2015 (E. Tselikh), 1 female (ZISP).

[GG] • Inchen-si, Jung-gu, Muui-dong, 37°23′46.09″N, 126°24′36.38″E, Malaise trap, 14–27.IX.2017 (Hyung-Keun Lee), 1 male (SMNE).

[GN] • Sancheong-gun, Chahwang-myeon, Mt. Hwangmae, (30 km NNW Jinju, forest, h = 800 m), 12.VI.2002 (S. Belokobylskij) (SMNE) • same locality, 29.VI.2002 (S. Belokobylskij), 1 female (ZISP) • Namhae-gun, Namhae-eup, Asan-ri, 34°51′06.7″N, 127°51′31″E, 19.VI.2022 (S. Belokobylskij), 1 female (ZISP).

[GW] • Jeongseon-gun, Imgye-myeon, Jikwon-ri, Mt. Seokbyeong, Malaise trap, 3.VIII–19.IX.2002 (Deokseo Ku), 1 male (SMNE) • Goseong-gun, Ganseong-eup, Jinbu-ri, Hyangrobong-peak, (DMZ), 13.VI.1992 (J.-W. Lee), 1 female, 2 males (SMNE) • Inje-gun, Girin-myeon, Jindong-ri, Mt. Jeombong, (Gombaeryeong), 38°1′58.52″N, 128°27′54.19″E, 14.IX–16.X.2017 (Hyung-Keun Lee), 1 female (SMNE) • Taebaek-shi, Mt. Taebaek, 13.VIII.1989 (S.-M. Ryu), 1 female (paratype) (SMNE); Mt. Taebaek, 23.VI.1989 (Deokseo Ku), 1 male (SMNE).

[JN] • Gurye-gun, Toji-myeon, Bangok-gil, Mt. Jiri, Nogodan, 35°17′37.2″N, 127°31′55.6″E, Malaise trap, 10.VII–11.IX.2001 (Deokseo Ku), 5 females (SMNE, ZISP).

##### Hosts.

Unknown.

##### Distribution.

Korean peninsula; Russia (southern Far East), Japan.

##### Remarks.

J. Papp (1987) recorded the species Colastes (Colastes) affinis (Wesmael, 1838) in the fauna of the Korean peninsula. However, during visit to the Hungarian Natural History Museum in 2000, the first author studied and determined that the Korean specimens belong actually to the East Palaearctic C. (C.) interdictus Belokobylskij, 1998. These corrections and new information were later published by Papp (2003: 118). Thus, the species C. (C.) affinis is here excluded from the fauna of the Korean peninsula.

#### ﻿ Colastes (Colastes) pubicornis

Taxon classificationAnimaliaHymenopteraBraconidae

(Thomson, 1892)

F0E56BC0-FAC0-5F58-BD4D-BEDE78FAD7FE

Exothecus pubicornis Thomson, 1892: 1699.
Colastes pubicornis : Shenefelt 1975: 1122; Belokobylskij and Tobias 1986: 53; Papp 2003: 118.
Xenarcha (Xenarcha) pubicornis : Papp 1987: 161.
Colastes (Colastes) pubicornis : Belokobylskij, 1998: 139; Ku et al. 2001: 154; Yu et al. 2016; Belokobylskij et al. 2019: 281.

##### Material examined.

South Korea: [GW] • Jeongseon-gun, Nam-myeon, Mungok-ri, Jamiwon (Doowibong-peak), 2.IX.2000 (collector unknown), 1 female (SMNE).

[GG] • Mt. Yumyeong, Okcheon-myeon, Yangpyeonggun, Gyeonggi-do, 14. VI. 1992 (collector unknown), 1 male (SMNE).

##### Hosts.

**Diptera**: *Chirosiahistricina* (Rondani, 1886) (Anthomyiidae).

##### Distribution.

Korean peninsula; Europe (rarely), Russia (European part, Siberia, Far East), China (Taiwan), Japan.

##### Remarks.

This species was also recorded from North Korea as *Colastesflavitarsis* Thomson (Papp, 1992). Papp (2003: 118) used later the correct determination of this species.

#### ﻿ Colastes (Colastes) semiflavus

Taxon classificationAnimaliaHymenopteraBraconidae

Belokobylskij & Ku sp. nov.

5B74961C-1B82-5CEA-AD04-DF663D1505E3

https://zoobank.org/70A45341-B4D4-4E33-BEFF-A03F2234AF97

Figs 3 , 4

##### Type material.

***Holotype*** • female, “Korea (GN) [= Gyeongsangnam-do]. Namhae-gun, Seo-myeon, Nogu-ri, Temple Mangunsa, 15.X–14.XI.2022 (Malaise trap), Deokseo Ku, Jaehyeon Lee, Hyojin Jeong (NIBR).

##### Description.

**Female**. ***Body*** length 2.8 mm; fore wing length 2.9 mm. ***Head*** width 1.8× its medial length (dorsal view), 1.2× wider than mesoscutum. Temple behind eye weakly convex in anterior 1/2 and distinctly curvedly narrowed in posterior 1/2. Transverse diameter of eye 1.6× longer than temple (dorsal view). Ocelli small, arranged in triangle with base 1.2× its sides. POL 1.5× Od, 0.8× OOL. Eye oval, glabrous, 1.25× as high as broad (lateral view). Malar space ~ 0.4× height of eye, almost equal to basal width of mandible. Malar suture indistinct. Face width 1.4× height of face and clypeus combined, equal to height of eye. Hypoclypeal depression circular, its width 1.1× distance from edge of depression to eye, 0.4× width of face. Head distinctly and almost linearly narrowed below eyes. ***Antenna*** weakly thickened, almost filiform, 28-segmented, ~ 1.2× longer than body. First flagellar segment 3.3× longer than its apical width, 1.2× longer than second segment. Penultimate segment 2.3× longer than wide, ~ 0.6× as long as first segment, 0.9× as long as apical segment; the latter acuminate apically and with short ‘spine’.

**Figure 3.**
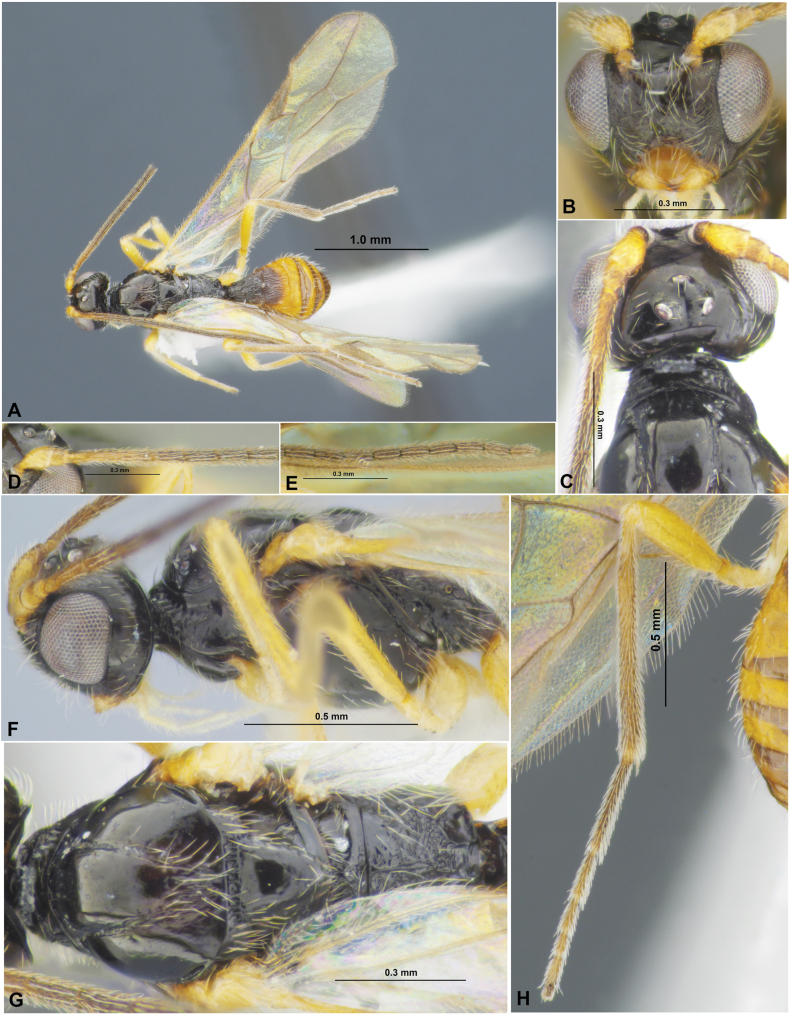
Colastes (Colastes) semiflavus sp. nov., female, holotype **A** habitus, dorsal view **B** head, front view **C** head and anterior part of mesosoma, dorsal view **D** basal segments of antenna **E** apical segments of antenna **F** head and mesosoma, lateral view **G** mesosoma, dorsal view **H** hind leg.

***Mesosoma*** 1.9× longer than its height. Pronotum dorsally with shallow and sculptured transverse groove, with weak transverse pronotal carina. Mesoscutum highly and curvedly elevated above prothorax. Notauli complete, deep in anterior 0.6, shallow in posterior 0.4, crenulate anteriorly. Prescutellar depression short, distinctly crenulate, ~ 0.2× as long as scutellum. Scutellum without transverse furrow and smooth posteriorly. Precoxal sulcus absent. ***Wings.*** Fore wing 2.7× longer than maximum width. Pterostigma relatively wide, 4.2× its maximum width, 0.8× as long as metacarp (1-R1). Radial vein (r) arising weakly before middle of pterostigma, from its basal ~ 0.45. Second radial abscissa (3-SR) 2.8× longer than first abscissa (r) and forming very obtuse angle with it, 0.5× as long as the straight third abscissa (SR1), 1.3× longer than first radiomedial vein (2-SR). Recurrent vein (m-cu) distinctly antefurcal. Second radiomedial (submarginal) cell not narrowed distally, its length 2.6× maximum width, 1.9× longer than brachial (subdiscal) cell. First abscissa of medial vein (1-SR+M) weakly sinuate. Distance (1-CU1) from nervulus (cu-a) to basal vein (1-M) 0.6× nervulus (cu-a) length. Parallel vein (CU1a) arising from posterior 0.4 of distal margin (3-CU1) of brachial (subdiscal) cell. Brachial (subdiscal) cell distinctly widened distally. In hind wing, first abscissa of mediocubital vein (M+CU) ~ 0.9× as long as second abscissa (1-M). First abscissa of costal vein (C+SC+R) approx. as long as second abscissa (1-SC+R). Recurrent vein (m-cu) long, unsclerotised, weakly antefurcal, weakly curved. ***Legs***. Hind femur 5.0× longer than wide. Inner spur of hind tibia ~ 0.2× as long as hind basitarsus. Hind tarsus as long as hind tibia. Hind basitarsus 0.7× as long as combined length of second to fifth segments. Second tarsal segment of hind leg 0.45× as long as basitarsus, 1.7× as long as fifth segment (without pretarsus).

**Figure 4.**
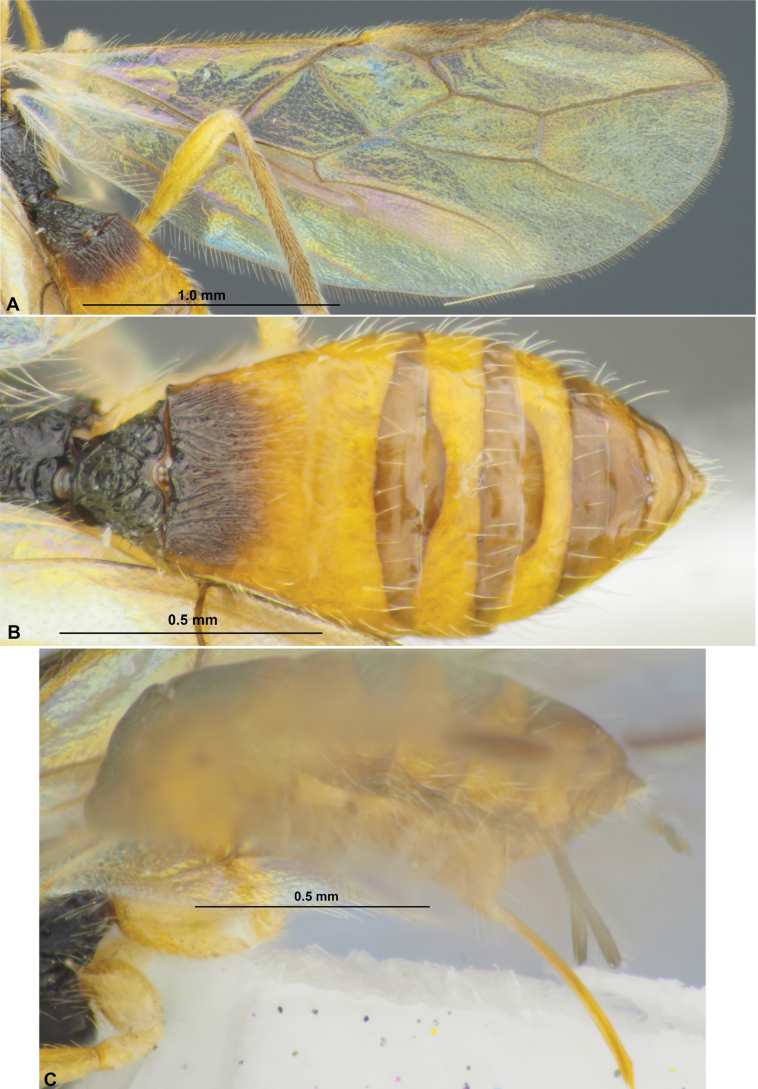
Colastes (Colastes) semiflavus sp. nov., female, holotype **A** wings **B** metasoma, dorsal view **C** metasoma and ovipositor, lateral view.

***Metasoma*** ~ 0.9× as long as head and mesosoma combined. First tergite comparatively slender, evenly and almost linearly widened from base to apex, with very weak spiracular tubercles, with dorsal carinae distinct in anterior 0.3 and connected subbasally by additional transverse carina. Length of first tergite 1.25× its anterior width, anterior width 1.7× its posterior width. Second suture distinct, wide, almost straight, rugulose. Medial length of second tergite 0.8× its anterior width, 1.3× length of third tergite. Setose part of ovipositor sheath 0.2× as long as metasoma, almost as long as first tergite, almost equal to hind basitarsus, ~ 0.1× as long as fore wing.

***Sculpture and pubescence.*** Vertex and temple smooth; face densely granulate in lateral lower 0.6, smooth or almost smooth on remaining part. Mesoscutum mainly smooth, with striation in medio-posterior 0.4; scutellum and mesopleuron smooth. Metapleuron widely smooth in anterior 0.5–0.6, rugulose-reticulate on remaining posterior part. Propodeum mainly rugose-reticulate, smooth in narrow anterior part, with distinct medial carina in anterior 1/2, areola small and weakly delineated by carinae. Legs (including hind coxa) entirely smooth. First metasomal tergite entirely and densely rugose-striate; second tergite entirely distinctly striate, almost without reticulation; third tergite rugose-reticulate in anterior 0.3–0.4, remaining part smooth. Remaining tergites smooth. Mesoscutum mainly glabrous, with sparse, semi-erect, short and pale setae arranged along notauli and in medio-posterior area. Hind tibia dorsally with short, dense and semi-erect brownish setae, length of these setae ~ 0.5× as long as maximum width of hind tibia.

***Colour.*** Head, mesosoma, first and anterior 1/2 of second metasomal tergites black; remaining part of metasoma yellow to brownish yellow. Antenna mainly dark brown to black, two basal segments yellowish brown with faint infuscation. Palpi pale yellow. Legs mainly yellow, hind tibia in distal 1/2 and hind tarsus brown. Wings hyaline. Pterostigma pale brown.

**Male**. Unknown.

##### Discussion.

This new species is very similar to C. (C.) ussuricus Belokobylskij, 1996, but differs from later by having the face width 1.4× height of face and clypeus combined (1.1–1.2× in *C.ussuricus*), antenna 28-segmented (32–35-segmented in *C.ussuricus*), tergites behind third tergite entirely smooth (fourth in basal 1/2 and fifth basally striate in *C.ussuricus*), metasoma in posterior 1/2 yellow to brownish yellow (metasoma black or sometimes partly dark reddish brown in *C.ussuricus*).

Colastes (C.) semiflavus sp. nov. is also close to C. (C.) interdictus Belokobylskij, 1998, but differs from later by having the face densely granulate in lower 0.6 (in lower 0.3 in *C.interdictus*), the first flagellar segment 3.3× longer than its apical width (4.0–5.0× in *C.interdictus*), penultimate segment of antenna 2.3× longer than wide (3.0× in *C.interdictus*), prescutellar depression short (relatively long in *C.interdictus*), radial vein (r) of fore wing arising from basal 0.45 of pterostigma (from basal 0.3 of pterostigma in *C.interdictus*), third metasomal tergite rugose-reticulate in anterior 0.3–0.4 (third tergite entirely smooth or only sometimes with short striae basally in *C.interdictus*), metasoma yellow to brownish yellow in posterior 0.5–0.6 (pale reddish brown behind first tergite in *C.interdictus*), and hind tibia in distal 1/2 and hind tarsus brown (hind legs entirely yellow in *C.interdictus*).

##### Etymology.

Named from the combination of the Latin words *semi* (half) and *flavus* (yellow) because metasoma is yellow in its posterior half.

##### Distribution.

Korean peninsula.

#### ﻿* Colastes (Colastes) sylvicola

Taxon classificationAnimaliaHymenopteraBraconidae

Belokobylskij, 1998

1C275643-98CA-55A6-B68A-9A870967C81E

Colastes (Colastes) sylvicola Belokobylskij, 1998: 141; Yu et al. 2016; Belokobylskij et al. 2019: 281.

##### Material examined.

South Korea: [GB] • Ulreung-gun, Buk-myeon, Nari-ri, Nariryeong, sweeping, 29.VII.2001 (collector unknown), 1 female (NIBR).

##### Hosts.

Unknown.

##### Distribution.

*Korean peninsula; Russia (southern Far East), Japan (Kyushu).

#### ﻿* Colastes (Colastes) ussuricus

Taxon classificationAnimaliaHymenopteraBraconidae

Belokobylskij, 1996

11305452-3CBB-54C0-9B5C-CFAA396FD798

Colastes (Colastes) ussuricus Belokobylskij, 1996: 1667; 1998: 141; Yu et al. 2016; Belokobylskij et al. 2019: 281.

##### Material examined.

South Korea: [GB] • Uljin-gun, Buk-myeon, Deokgu-ri, Huinmoki, 6.V.2000 (collector unknown), 1 female (NIBR).

##### Hosts.

Unknown.

##### Distribution.

*Korean peninsula; Russia (southern Far East), Japan (Kyushu).

#### ﻿Genus Colastinus

Taxon classificationAnimaliaHymenopteraBraconidae

Belokobylskij, 1984

B7471CF0-839D-5291-B1E0-39F97A55B0ED

##### Type species.

*Colastinuscrustatus* Belokobylskij, 1984.

#### ﻿* Colastinus crustatus

Taxon classificationAnimaliaHymenopteraBraconidae

Belokobylskij, 1984

B9E53AA1-D5AE-5CE6-811C-68387030C0FF

Fig. 9

Colastinus crustatus Belokobylskij, 1984: 1022; 1994: 73; 1998: 159; Yu et al. 2016; Belokobylskij et al. 2019: 282.

##### Material examined.

South Korea: [GB] • Yeongyang-gun, Ilwol-myeon, Mt. Ilwolsan, 36°48'29″N, 129°05'25″E, 7.VII.2015 (E. Tselikh), 1 female (NIBR).

##### Hosts.

Unknown.

##### Distribution.

*Korean peninsula; Russia (southern Far East).

#### ﻿Genus Xenarcha

Taxon classificationAnimaliaHymenopteraBraconidae

Foerster, 1863

BB3E07C8-26B0-55FD-98BC-4E9E1F00F256

##### Type species.

Rogas (Colastes) lustrator Haliday, 1836.

#### ﻿ Subgenus Pseudophanomeris

Taxon classificationAnimaliaHymenopteraBraconidae

Belokobylskij, 1984 syn. nov.

77E151C0-F1DF-560A-BF48-99FD2EF68844

##### Type species.

Colastes (Pseudophanomeris) unicolor Belokobylskij, 1984.

#### ﻿* Xenarcha (Pseudophanomeris) insularis

Taxon classificationAnimaliaHymenopteraBraconidae

(Belokobylskij, 1984) comb. nov.

3FEB26B7-FD0A-5464-A243-C4E5AF8E1E5A

Colastes (Pseudophanomeris) insularis Belokobylskij, 1984: 1024; 1994: 70; 1998: 145; Yu et al. 2016; Belokobylskij et al. 2019: 281.

##### Material examined.

South Korea: [GG] • Gwangju-si, Toechon-myeon, Gwaneum-ri, 37°26′43.6″N, 127°19′55.26″E, Malaise trap, 24.V–6.VI.2017 (Hyung-Keun Lee), 1 female (SMNE).

[GB] • Cheongsong-gun, Budong-myeon, Sangui-ri, Mt. Juwang, sweeping, 20.VII.1999 (Jungsuk Park), 1 male (NIBR).

##### Hosts.

Unknown.

##### Distribution.

*Korean peninsula; Russia (southern Far East), Japan.

#### ﻿ Xenarcha (Pseudophanomeris) pilosa

Taxon classificationAnimaliaHymenopteraBraconidae

(Belokobylskij, 1984) comb. nov.

7626722B-BEDA-5333-902B-A57D56A24C39

Colastes (Pseudophanomeris) pilosa Belokobylskij, 1984: 1024; 1994: 70; 1998: 145; Yu et al. 2016; Belokobylskij et al. 2019: 281.
Xenarcha (Pseudophanomeris) pilosa : Papp 1987: 160.

##### Material recorded.

This species was previously recorded from North Korea (Papp 1987) • “Kangwon Province, Kum-gang-san, 12.X.1978, No 488”, 2 females (HNHM).

##### Hosts.

Unknown.

##### Distribution.

Korean peninsula; Czech Republic, Ukraine, Russia (European part, southern Fart East), Japan.

#### ﻿ Subgenus Shawiana

Taxon classificationAnimaliaHymenopteraBraconidae

van Achterberg, 1983

C4417E90-1FE0-565F-9672-156A84D58EBD

##### Type species.

*Exothecuslaevis* Thomson, 1892.

#### ﻿ Xenarcha (Shawiana) attonita

Taxon classificationAnimaliaHymenopteraBraconidae

(Papp, 1987) comb. nov.

75B2081D-B448-50DA-AC70-A718CD8A5CD8

Shawiana attonita Papp, 1987: 173; Yu et al. 2016.
Colastes (Shawiana) attonitus : Belokobylskij 1998: 148.

##### Material recorded.

North Korea • “Korea, Prov. North Pyongan, Mt. Myohyang-san (first label). ‘’Hotel. 14 VIII 1982, leg. Beron et Porov, No. 11” (second label), 1 female (holotype) (HNHM, Nº.7077) (examined).

##### Hosts.

Unknown.

##### Distribution.

Korean peninsula.

#### ﻿* Xenarcha (Shawiana) catenator

Taxon classificationAnimaliaHymenopteraBraconidae

(Haliday, 1836) comb. nov.

BB386D89-4257-5701-9E73-AFDF612024CD

Rhogas (Colastes) catenator Haliday, 1836: 93.
Phanomeris catenator : Shenefelt 1975: 1130; van Achterberg 1983: 345.
Shawiana catenator : van Achterberg 1983: 345; Yu et al. 2016.
Colastes catenator : Belokobylskij and Tobias 1986: 52; Belokobylskij 1994: 71; 1998: 152.
Colastes (Shawiana) catenator : Belokobylskij et al. 2019: 282.

##### Material examined.

South Korea: [GN] • Sancheong-gun, Chahwang-myeon, Mt. Hwangmae, (30 km NNW Jinju, forest, h = 800 m), 12.VI.2002 (S. Belokobylskij), 1 female (NIBR).

##### Hosts.

**Hymenoptera**: *Fenellanigrita* (Westwood), *Fenusadohrnii* (Tischbein), *F.pumila* Leach, *F.pusilla* Leach, *F.rubi* Boie, *F.ulmi* Sundevall, *Heterarthrusaceris* (Kaltenbach), *H.vagans* Fallen, *Messahortulana* (Klug), *M.nana* (Klug), *Metallusalbipes* (Cameron), *M.pumilus* (Klug), *Parnatenella* (Klug), *Profenusapygmaea* (Klug), *Scolioneurabetuleti* (Klug) (Tenthredinidae). **Lepidoptera**: *Eriocraniasemipurpurella* (Stephens) (Eriocraniidae); *Phyllonorycterquercifoliella* (Zeller) (Gracillariidae).

##### Distribution.

*Korean peninsula; Western Europe, Georgia, Russia (European part, Siberia, Far East), Mongolia, Japan.

#### ﻿* Xenarcha (Shawiana) foveolator

Taxon classificationAnimaliaHymenopteraBraconidae

(Thomson, 1892) comb. nov.

C8101EF1-B6BC-5CB7-B00D-DDA3902E23D4

Exothecus foveolator Thomson, 1892: 1698.
Colastes foveolator : Shenefelt 1975: 1119; Belokobylskij and Tobias 1986: 57.
Shawiana foveolator : van Achterberg 1983: 348; Yu et al. 2016.
Colastes (Shawiana) foveolator : Belokobylskij 1998: 145; Belokobylskij et al. 2019: 282.

##### Material examined.

South Korea: [GN] • Goseong-gun, Hail-myeon, Suyang-ri, 34°58′34.8″N, 128°12′08.3″E, 18.VI.2022 (E. Tselikh), 1 female (NIBR).

##### Hosts.

**Hymenoptera**: *Blasticotomafiliceti* Klug (Blasticotomidae). **Lepidoptera**: *Phyllonorycteralpina* (Fray), *Ph.coryli* (Nicelli) (Gracillariidae)

##### Distribution.

*Korean peninsula; Germany, Sweden, Finland, Russia (European part, Siberia, Far East), Japan.

#### ﻿ Xenarcha (Shawiana) laevis

Taxon classificationAnimaliaHymenopteraBraconidae

(Thomson, 1892) comb. nov.

BB9C2245-BB66-5EBA-AFE5-6A4512372D57

Fig. 11

Exothecus laevis Thomson, 1892: 1699.
Colastes laevis : Shenefelt 1975: 1120 Belokobylskij and Tobias 1986: 53.
Shawiana laevis : van Achterberg 1983: 343; Papp 1987: 160; 1992: 66; Yu et al. 2016.
Colastes (Shawiana) laevis : Belokobylskij 1998: 147; Belokobylskij et al. 2019: 282.

##### Material examined.

South Korea: [JN] • Gurye-gun, Toji-myeon, Bangok-gil, Mt. Jiri, Nogodan, 35°17′37.2″N, 127°31′55.6″E, Malaise trap, 10.VII–11.IX.2001 (Deokseo Ku), 1 female (SMNE).

##### Hosts.

**Hymenoptera**: *Euuramucronata* (Hartig), *Fenusadohrnii* (Tischbein), *Heterarthrus aceris* (Kaltenbach), *H.microcephalus* (Klug), *H.vagans* Fallen, *H.wuestneii* (Konow), *Pontaniaglaucae* Kopelke, *P.lapponicola* Kopelke, *P.nigricantis* Kopelke, *P.vesicator* Bremi, *Scolioneurabetuleti* (Klug), *S.vicina* Konow (Tenthredinidae).

##### Distribution.

Korean peninsula; Europe (widely), Russia (European part, Far East), Turkey, Iran, Mongolia.

#### ﻿ Xenarcha (Shawiana) nupta

Taxon classificationAnimaliaHymenopteraBraconidae

(Papp, 1983) comb. nov.

A843B30E-0CD8-5FDF-AC2E-B1BD6125812E

Colastes nuptus Papp, 1983: 447.
Shawiana nupta : Papp 1987: 160; Yu et al. 2016.
Colastes (Shawiana) nuptus : Belokobylskij 1998: 152; Belokobylskij et al. 2019: 282.

##### Material examined.

South Korea: [GN] • Geochang-gun, Sinwon-myeon, Waryong-ri, Malaise trap, 2–16.VII.2022 (Deokseo Ku, Jaehyeon Lee, Hyojin Jeong), 1 female (SMNE).

##### Hosts.

Unknown.

##### Distribution.

Korean peninsula; Mongolia, Russia (southern Far East).

#### ﻿ Xenarcha (Shawiana) orientalis

Taxon classificationAnimaliaHymenopteraBraconidae

(Belokobylskij, 1998) comb. nov.

8162027C-5D45-56B7-8B2B-8EA6B819E819

Colastes (Shawiana) orientalis Belokobylskij, 1998: 152; Ku et al. 2001: 155; Belokobylskij et al. 2019: 282.
Shawiana orientalis : Yu et al. 2016.

##### Material examined.

South Korea: [GB] • Andong-si, Bukhu-myeon, Daehyeon-ri, Malaise trap, 3–18.V.2022.(Gi-Myon Kwon), 1 female (SMNE) • Sonsan-gun, Suryun, Bongyang-ri, 9.VI.1992 (Deokseo Ku), 1 female (paratype) (SMNE).

[GN] • Namhae-gun, Namhae-eup, Simcheon-ri, Simcheon, light trap, 26–27. IX.1998 (Jesik Jeon), 1 female (SMNE).

[GW] • Sokcho-si, Seolak-dong, 12.VI.1992 (Deokseo Ku), 1 female (SMNE).

##### Hosts.

Unknown.

##### Distribution.

Korean peninsula; Russia (Siberia, southern Far East).

#### ﻿* Xenarcha (Shawiana) rupicola

Taxon classificationAnimaliaHymenopteraBraconidae

(Belokobylskij, 1998) comb. nov.

1A2A779B-9EAC-5AB7-8B4C-FBE8DD860AFB

Colastes (Shawiana) rupicola Belokobylskij, 1998: 147; Belokobylskij et al. 2019: 282.
Shawiana rupicola : Yu et al. 2016.

##### Material examined.

South Korea: [JN] • Gurye-gun, Toji-myeon, Bangok-gil, Mt. Jiri, Nogodan, 35°17′37.2″N, 127°31′55.6″E, Malaise trap, 10.VIII–11.IX.2001 (Deokseo Ku), 1 female (NIBR).

##### Hosts.

Unknown.

##### Distribution.

*Korean peninsula; Russia (southern Far East).

### ﻿SubgenusXenarcha s. str.

#### ﻿ Xenarcha (Xenarcha) effecta

Taxon classificationAnimaliaHymenopteraBraconidae

(Papp, 1972) comb. nov.

85C81A1F-05A4-58D8-8B92-4FFAFBF292DE

Exothecus effectus Papp, 1972: 323.
Colastes effectus : Shenefelt 1975: 1119.
Xenarcha effecta : van Achterberg 1983: 349.
Colastes (Shawiana) effectus : Belokobylskij 1990: 37; 1994: 71.
Colastes (Xenarcha) effectus : Belokobylskij 1998: 154; Ku et al. 2001: 154.
Colastes (Fungivenator) effectus : van Achterberg and Shaw 2008: 1854; Yu et al. 2016; Belokobylskij et al. 2019: 281.

##### Material examined.

South Korea: [GN] • Jinju, Gajwa, 28.IX.1993 (Deokseo Ku), 1 female (SMNE); • Sancheong-gun, Chahwang-myeon, Mt. Hwangmae, (30 km NNW Jinju, forest, h = 800 m), 16.VI.2002 (S. Belokobylskij), 1 female (ZISP).

[GB] • Yeongcheon-si, Hyeonseo-myeon, Galjeon-ri, Mt. Bohyeon, sweeping, 4.IX.1998 (Jungsuk Park), 1 female (SMNE).

##### Hosts.

Unknown.

##### Distribution.

Korean peninsula; Russia (European part, southern Far East), China, Japan.

##### Remarks.

This species was included in the subgenusFungivenator van Achterberg & Shaw, 2008 of the genus *Colastes* Haliday (van Achterberg and Shaw 2008). However the material of our study from China, Korea, and the Russian Far East distinctly shows that the members of this species have a distinct and deep round pronope, which together with some other characters (complete notauli, radial vein (r) of the fore wing arising almost from middle of pterostigma) demonstrate that it belongs to Xenarcha (subgenus Xenarcha).

#### ﻿* Xenarcha (Xenarcha) ivani

Taxon classificationAnimaliaHymenopteraBraconidae

(Belokobylskij, 1986) comb. nov.

E4375868-67B2-5BA7-A5EF-F3667D6BAB8F

Colastes ivani Belokobylskij, 1996: 68.
Colastes (Shawiana) ivani : Belokobylskij 1994: 72; 1998: 156.
Xenarcha ivani : Yu et al. 2016.
Colastes (Xenarcha) ivani : Belokobylskij et al. 2019: 282.

##### Material examined.

South Korea: [GN] • Namhae-gun, Seo-myeon, Nogu-ri, Temple Mangunsa, Malaise trap, 27.VII–13.VIII.2022 (Deokseo Ku, Jaehyeon Lee, Hyojin Jeong), 1 female (NIBR) • Namhae-gun, Namhae-eup, Asan-ri, 34°51′06.7″N, 127°51′31″E, 19.VI.2022 (S. Belokobylskij), 1 male (SMNE) • Sancheong-gun, Chahwang-myeon, Mt. Hwangmae, (30 km NNW Jinju, forest, h = 800 m), 10.VII.2002 (S. Belokobylskij), 1 male (ZISP).

##### Hosts.

Unknown.

##### Distribution.

*Korean peninsula; Russia (southern Far East).

#### ﻿ Xenarcha (Xenarcha) pacificoformis

Taxon classificationAnimaliaHymenopteraBraconidae

Belokobylskij & Ku sp. nov.

1C909280-8EFA-57D3-B033-191E7D5E2B75

https://zoobank.org/583B493F-ACE4-4D6E-A534-A1FB1BD580AC

Figs 5 , 6

##### Type material.

***Holotype*** • female, South Korea, Gyeonggi-do [GG], Yangpyeong-gun, Okcheon-myeon, Mt. Yumyeong, 14.VI.1992 (collector unknown) (NIBR). ***Paratypes***: South Korea. [**GG**] • Yangpyeong-gun, Okcheon-myeon, Yongcheon-ri, Mt. Yongmun, sweeping, 28.VII.2000 (Tae-Ho An), 1 female (SMNE) • Suwon-si, Gwonseon-gu, Seodun-dong, Mt.Yeogi, 5.VIII.1997 (June-Yeol Choi), 1 female (SMNE) • Yangpyeong-gun, Okcheon-myeon, Yongcheon-ri, Mt. Yongmun, sweeping, 28.VII.2000 (Tae-Ho An), 1 male (SMNE). [**GW**] • Cheolwon-gun, Galmal-eup, Naedae-ri, 14.VI.1992 (collector unknown), 1 female (SMNE) • Sokcho-si, Seolak-dong, 12.VI.1992 (Deokseo Ku), 1 female (SMNE) • Gangreung-si, Yeongok-myeon, Sinwang-ri Sogeum River (Mt. Odae), 3.VIII.1998 (collector unknown), 1 male (SMNE). [**GB**] • Yeongyang-gun, Inwol-myeon, Mt. Inwol, 36°48'29″N, 129°05'25″E, 14.VII.2023 (E. Tselikh), 1 female (ZISP) • Seongju-gun, Suryun-myeon, Baekun-ri, Baekunbunso, Mt.Gaya, sweeping, 22.VIII.2000 (Tae-Ho An), 1 female (SMNE) • Ulleung-gun, Ulleung-eup, Sadong-ri, San35 Nari-Seonginbong No.15, sweeping, 10.IX.2017, (Deokseo Ku), 1 male (SMNE) • Cheongsong-gun, Budong-myeon, Sangui-ri, Mt. Juwang, 20.VII.1999 (Juhwan Son, Gyeongryeon Han), 2 males (SMNE) • Cheongsong-gun, Budong-myeon, Sangui-ri, Mt. Juwang, 20.VII.1999 (Juhwan Son, Gyeongryeon Han), 2 males (SMNE, ZISP). [**GN**] • Geochang-gun, Wicheon-myeon, Janggi-ri, Malaise trap, 16.X–30.XI.2015 (Tae-Ho An), 1 female (SMNE) • Goseong-gun, Gaecheon-myeon, Bukpyeong-ri, Mt.Yeonhwa, Temple Okcheon, sweeping, 22.V.1999 (Seongnam Kim), 1 female (SMNE) • Sancheong-gun, Chahwang-myeon, Mt. Hwangmae, (30 km NNW Jinju, forest, meadow, h = 800 m), 12.VI.2002.(S. Belokobylskij), 1 female (ZISP) • same label, but 16.VI.2002, 2 females (SMNE, ZISP) • same label, but 29.VI.2002, 1 male (ZISP) • same label, but 10.VII.2002, 1 male (ZISP).

**Figure 5.**
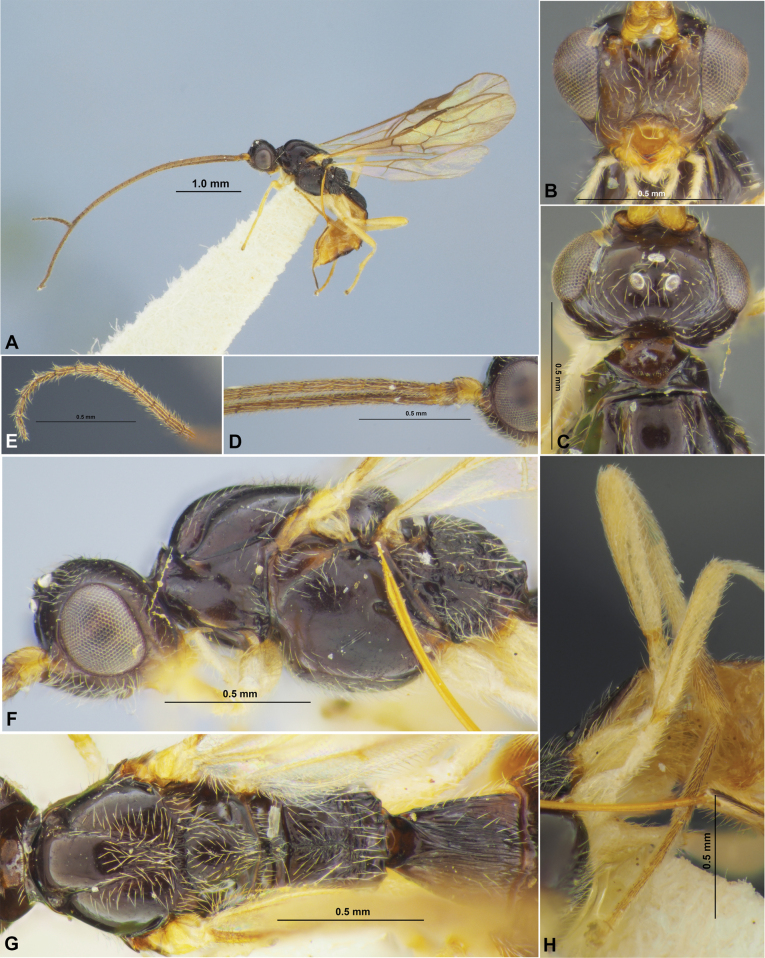
Xenarcha (Xenarcha) pacificoformis sp. nov., female, holotype **A** habitus, lateral view **B** head, front view **C** head and anterior part of mesosoma, dorsal view **D** basal segments of antenna **E** apical segments of antenna **F** head and mesosoma, lateral view **G** mesosoma and first metasomal tergite, dorsal view **H** hind leg.

##### Description.

**Female**. ***Body*** length 3.1–4.1 mm; fore wing length 3.0–4.1 mm. ***Head*** width 1.8–1.9× its medial length (dorsal view), ~ 1.3× wider than mesoscutum. Temple strongly curvedly narrowed behind eye. Transverse diameter of eye 1.5–1.6× longer than temple (dorsal view). Ocelli small, arranged almost in equilateral triangle. POL 1.0–1.2× Od, 0.45–0.50× OOL. Eye oval, glabrous, l.3× as high as broad (lateral view). Malar space 0.3–0.4× height of eye, 1.0–1.3× basal width of mandible. Malar suture absent or almost absent. Face width 1.2–1.3× height of face and clypeus combined, almost equal to height of eye. Hypoclypeal depression circular, its width 0.8–1.0× distance from edge of depression to eye, ~ 0.4× width of face. Head distinctly and almost linearly narrowed below eyes. ***Antenna*** rather slender, filiform or weakly setiform, 34–36-segmented, ~ 1.2× longer than body. First flagellar segment 3.3–3.7× longer than its apical width, 1.2–1.3× longer than second segment. Penultimate segment 2.7–2.8× longer than wide, ~ 0.5× as long as first segment, ~ 0.8× as long as apical segment; the latter acuminate apically and with short ‘spine’.

**Figure 6.**
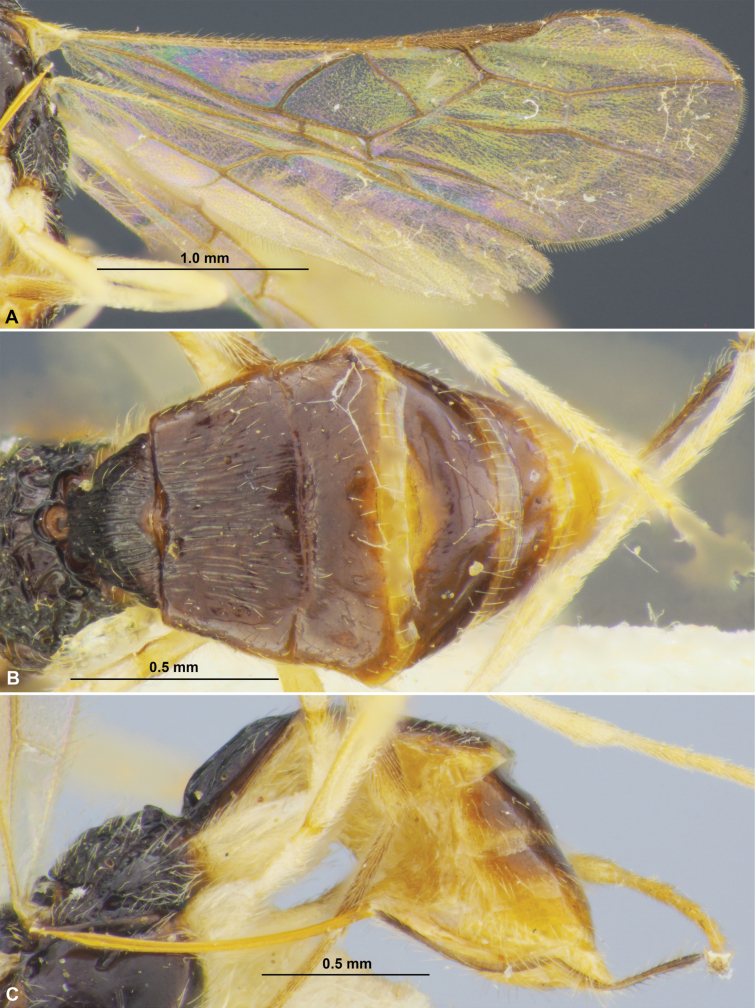
Xenarcha (Xenarcha) pacificoformis sp. nov., female, holotype **A** wings **B** metasoma, dorsal view **C** propodeum, metasoma and ovipositor, lateral view.

***Mesosoma*** 1.8–2.0× longer than its height. Pronotum dorsally with large, deep, smooth round pit (pronope), with high and strongly curved medially transverse carina. Mesoscutum distinctly and curvedly elevated above prothorax. Notauli complete, rather deep, finely and sparsely crenulate. Prescutellar depression distinctly rugulose-crenulate, 0.25–0.30× as long as scutellum. Scutellum without transverse furrow and smooth posteriorly. Precoxal sulcus absent. ***Wings.*** Fore wing 2.8–3.0× longer than maximum width. Pterostigma narrow, 5.0–5.6× its maximum width, 0.8–0.9× as long as metacarp (1-R1). Radial vein (r) arising before middle of pterostigma, from its basal ~ 0.4. Second radial abscissa (3-SR) 2.5–3.0× longer than first abscissa (r) and forming obtuse angle with it, ~ 0.5× as long as the almost straight third abscissa (SR1), 1.2–1.4× longer than first radiomedial vein (2-SR). Recurrent vein (m-cu) distinctly antefurcal. Second radiomedial (submarginal) cell weakly narrowed distally, its length 2.5–2.7× maximum width, 1.7–2.0× longer than brachial (subdiscal) cell. First abscissa of medial vein (1-SR+M) weakly sinuate. Distance (1-CU1) from nervulus (cu-a) to basal vein (1-M) 1.2–1.3× nervulus (cu-a) length. Parallel vein (CU1a) arising from posterior 0.3 of distal margin (3-CU1) of brachial (subdiscal) cell. In hind wing, first abscissa of mediocubital vein (M+CU) 1.0–1.1× as long as second abscissa (1-M). First abscissa of costal vein (C+SC+R) 0.8–0.9× as long as second abscissa (1-SC+R). Recurrent vein (m-cu) long, unsclerotised, infuscate, antefurcal. ***Legs***. Hind femur 4.6–5.3× longer than wide. Inner spur of hind tibia ~ 0.2× as long as hind basitarsus. Hind tarsus approximately as long as hind tibia. Hind basitarsus 0.7–0.8× as long as combined length of second to fifth segments. Second tarsal segment of hind leg 0.45–0.50× as long as basitarsus, 1.4–1.7× as long as fifth segment (without pretarsus).

***Metasoma*** 0.9–1.1× longer than head and mesosoma combined. First tergite comparatively slender, evenly and linearly widened from base to apex, with very weak spiracular tubercles, with distinct dorsal carinae fused in basal one-third. Length of first tergite 1.2–1.3× its anterior width, anterior width 2.3–2.5× its posterior width. Second suture distinct, rather deep, almost straight, shortly crenulate or smooth. Medial length of second tergite ~ 0.7× its anterior width, 1.2–1.3× length of third tergite. Setose part of ovipositor sheath ~ 0.3× as long as metasoma, 1.0–1.1× as long as first tergite, 1.0–1.1× as long as hind basitarsus, 0.15–0.17× as long as fore wing.

***Sculpture and pubescence.*** Vertex, frons and temple smooth; face almost smooth in upper 1/2 and densely reticulate-granulate in lower 0.4–0.5. Mesoscutum mainly smooth, with striation in narrow area in medioposterior quarter; scutellum and mesopleuron smooth. Metapleuron widely smooth, rugulose-reticulate in posterior 0.2. Propodeum with or without medial carina in basal 1/2; without delineated areas, mainly rugulose-reticulate, smooth or almost smooth in anterior 0.2–0.4. Legs smooth. First metasomal tergite entirely and densely striate, without reticulation between striae; second tergite mainly striate, smooth or almost smooth laterally and in posterior 0.2–0.3; second suture smooth or with short crenulae; remaining tergites smooth. Mesoscutum mainly glabrous, with sparse, semi-erect or erect and long pale setae arranged along notauli and in medio-posterior area. Hind tibia dorsally with short, very dense and semi-erect pale setae.

***Colour.*** Body mainly black; metasoma behind first or second tergite dark reddish brown. Antenna mainly black, 2–3 basal segments brownish yellow with infuscation. Palpi pale yellow. Legs mainly yellow, hind tibia in distal 0.3 and hind tarsus more or less distinctly infuscate. Wings hyaline. Pterostigma brown.

**Male**. Body length 3.2–3.5 mm; fore wing length 2.9–3.4 mm. Antenna 34–37-segmented. Pterostigma of fore wing distinctly sclerotised, wide, dark brown, 3.8–4.5× its maximum width. Length of first tergite 1.2–1.4× its anterior width. Second suture usually crenulate. Medial length of second tergite 0.8–0.9× its anterior width, 1.3–1.5× length of third tergite. Hind leg sometimes darkened. Otherwise similar to female.

##### Discussion.

This new species is very similar to Xenarcha (Xenarcha) pacifica (Belokobylskij, 1998), comb. nov. (Fig. 8), but differs from later by having the strongly curved temple (less strongly curved in *X.pacifica*), the penultimate segment of antenna 2.7–2.8× longer than wide (2.0–2.3× in *X.pacifica*), pronotum dorsally with wide pronope and high curved transverse carina (with narrow pronope and without visible carina in *X.pacifica*), and the first metasomal tergite 1.2–1.3× longer than its anterior width (1.0–1.1× in *X.pacifica*).

##### Etymology.

Named after the related species *Xenarcha pacifica* and the Latin word *formis* (form) because it is similar to this species.

##### Distribution.

Korean peninsula.

#### ﻿ Xenarcha (Xenarcha) pacifica(Belokobylskij, 1998)ssp.brevisculpta

Taxon classificationAnimaliaHymenopteraBraconidae

Belokobylskij & Ku ssp. nov.

7F153CFA-7F5A-50CE-AA7E-D09F60929A69

https://zoobank.org/426F409D-8863-4ACF-8953-869A98ED32F5

Fig. 7

##### Type material.

***Holotype*** • female, South Korea, [GW], Taebaek-si, Mt. Taebaek, 13.VIII.1989 (Seungman Ryu) (NIBR). ***Paratypes*** • Same label as holotype. 5 females, 1 male (SMNE, ZISP) • same label, but 13.VIII.1989 (Wonyeong Choi), 1 female (SMNE).

##### Description.

**Female**. ***Body*** length 3.0–3.5 mm; fore wing length 3.4–3.8 mm. ***Head*** width 1.8–1.9× its medial length (dorsal view), ~ 1.2× wider than mesoscutum. Temple distinctly and convex-curvedly narrowed behind eye. Transverse diameter of eye 1.2–1.3× longer than temple (dorsal view). Ocelli small, arranged almost in equilateral triangle. POL 1.0–1.2× Od, 0.3–0.4× OOL. Eye oval, glabrous, 1.25–1.30× as high as broad (lateral view). Malar space 0.4–0.5× height of eye, 0.9–1.3× basal width of mandible. Malar suture very fine. Face width 1.1–1.2× height of face and clypeus combined, 1.0–1.1× height of eye. Hypoclypeal depression circular, its width almost equal to distance from edge of depression to eye, ~ 0.4× width of face. Head distinctly and almost linearly narrowed below eyes. ***Antenna*** rather slender, weakly setiform, 37–38-segmented, ~ 1.3× longer than body. First flagellar segment 3.5–3.8× longer than its apical width, 1.2–1.3× longer than second segment. Penultimate segment 2.3–2.6× longer than wide, ~ 0.5× as long as first segment, 0.8–0.9× as long as apical segment; the latter acuminate apically and with short ‘spine’.

**Figure 7.**
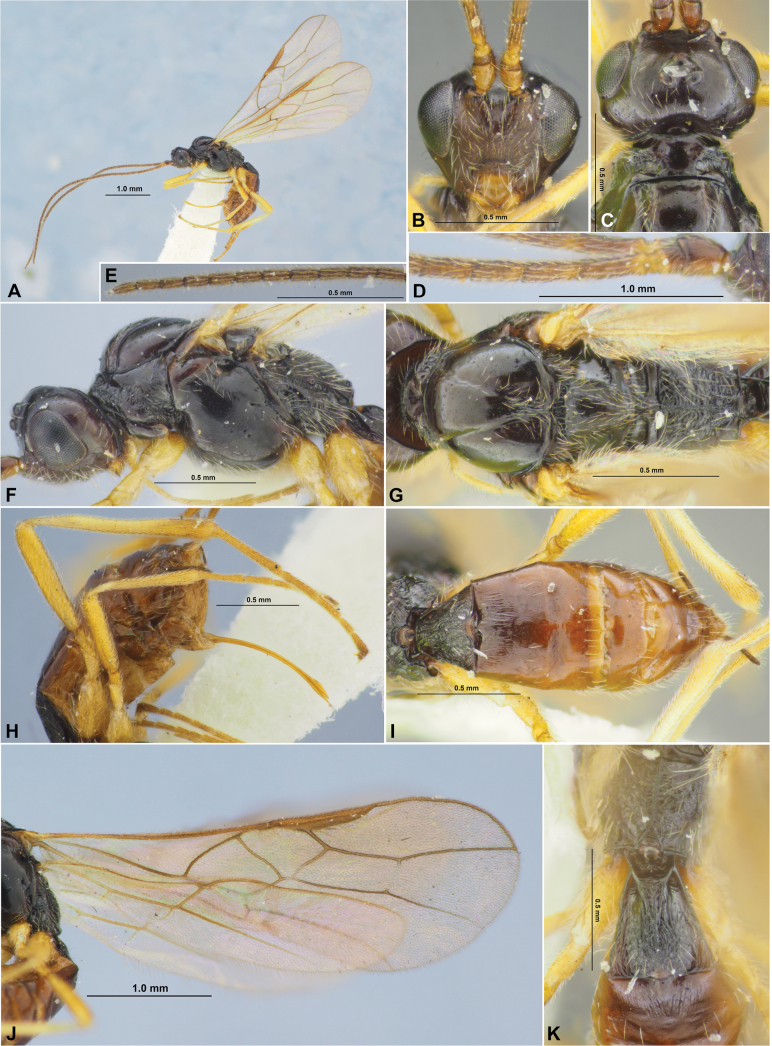
Xenarcha (Xenarcha) pacifica ssp. brevisculpta ssp. nov., female, holotype **A** habitus, lateral view **B** head, front view **C** head and anterior part of mesosoma, dorsal view **D** basal segments of antenna **E** apical segments of antenna **F** head and mesosoma, lateral view **G** mesosoma, dorsal view **H** metasoma and hind leg, lateral view **I** metasoma, dorsal view **J** wings **K** propodeum and anterior part of metasoma, dorsal view.

***Mesosoma*** 1.7–1.8× longer than its height. Pronotum dorsally with distinct, deep and smooth round pit (pronope), without transverse pronotal carina. Mesoscutum distinctly and curvedly elevated above prothorax. Notauli complete, rather deep, but more shallow posteriorly, smooth. Prescutellar depression short, coarsely rugulose-crenulate, 0.25× as long as scutellum. Scutellum without transverse furrow and smooth posteriorly. Precoxal sulcus completely absent. ***Wings.*** Fore wing 2.8–3.0× longer than maximum width. Pterostigma narrow, 5.0–5.3× its maximum width, 0.7–0.8× as long as metacarp (1-R1). Radial vein (r) arising distinctly before middle of pterostigma, from its basal ~ 0.3. Second radial abscissa (3-SR) 2.7–3.0× longer than first abscissa (r) and forming very obtuse angle with it, 0.5–0.6× as long as the straight third abscissa (SR1), 1.6–1.8× longer than first radiomedial vein (2-SR). Recurrent vein (m-cu) weakly antefurcal. Second radiomedial (submarginal) cell not narrowed distally, its length 2.6–2.8× maximum width, 1.7–1.9× longer than brachial (subdiscal) cell. Brachial (subdiscal) cell distinctly widened distally. First abscissa of medial vein (1-SR+M) sinuate. Distance (1-CU1) from nervulus (cu-a) to basal vein (1-M) 1.2–1.3× nervulus (cu-a) length. Parallel vein (CU1a) arising from posterior 0.4 of distal margin (3-CU1) of brachial (subdiscal) cell. In hind wing, first abscissa of mediocubital vein (M+CU) 1.0–1.1× as long as second abscissa (1-M). First abscissa of costal vein (C+SC+R) 0.6–0.7× as long as second abscissa (1-SC+R). Recurrent vein (m-cu) not long, mainly unsclerotised, infuscate basally, weakly antefurcal and weakly curved. ***Legs***. Hind femur 5.5–5.6× longer than wide. Inner spur of hind tibia ~ 0.2× as long as hind basitarsus. Hind tarsus approximately as long as hind tibia. Hind basitarsus ~ 0.6× as long as combined length of second to fifth segments. Second tarsal segment of hind leg 0.5–0.6× as long as basitarsus, 1.5–1.7× as long as fifth segment (without pretarsus).

***Metasoma*** 1.0–1.1× longer than head and mesosoma combined. First tergite comparatively slender, evenly and linearly widened from base to apex, with very weak spiracular tubercles, with distinct dorsal carinae fused in basal one-third by additional transverse carina. Length of first tergite 1.0–1.1 (rarely 1.15)× its anterior width, anterior width 2.0–2.3× its posterior width. Second suture weak, shallow, weakly curved, smooth. Medial length of second tergite 0.8–0.9× its anterior width, 1.6–1.7× length of third tergite. Setose part of ovipositor sheath ~ 0.2× as long as metasoma, usually as long as first tergite, 0.7–1.0× as long as hind basitarsus, ~ 0.1× as long as fore wing.

***Sculpture and pubescence.*** Vertex, frons and temple smooth; face mainly densely granulate, smooth or almost smooth medially in upper 0.3. Mesoscutum mainly smooth, with striation in narrow area in medioposterior quarter; scutellum and mesopleuron smooth. Metapleuron mainly coarsely transverse striate with rugosity, almost smooth in anterior 0.3. Propodeum entirely or almost entirely coarsely rugose-reticulate, with distinct medial carina in basal 0.6–0.7; without delineated areas. Legs mainly smooth, but hind coxa almost entirely rugose-reticulate. First metasomal tergite entirely and densely curvedly striate, with dense and coarse reticulation between striae; second tergite finely striate in anterior 0.3–0.5, smooth on remaining posterior part; remaining tergites entirely smooth. Mesoscutum mainly glabrous, with short, sparse and semi-erect pale setae arranged along notauli and in medio-posterior area. Hind tibia dorsally with short, very dense and semi-erect pale setae.

***Colour.*** Body mainly black; metasoma behind first tergite reddish brown or dark reddish brown. Antenna mainly dark brown or black, 2–3 basal segments faintly paler. Palpi pale yellow. Legs mainly yellow, hind tarsus faintly infuscate. Wings hyaline. Pterostigma brown.

**Male**. Body length 3.4 mm; fore wing length 3.2 mm. Antenna 36-segmented. Pterostigma of fore wing additionally sclerotised, dark brown, 3.2× its maximum width. Length of first tergite 1.4× its anterior width. Second suture finely and shortly crenulate. Second tergite striate-rugulose in anterior 0.7, its medial length 0.9× anterior width, 1.2× length of third tergite. Otherwise similar to female.

##### Discussion.

**Figure 8.**
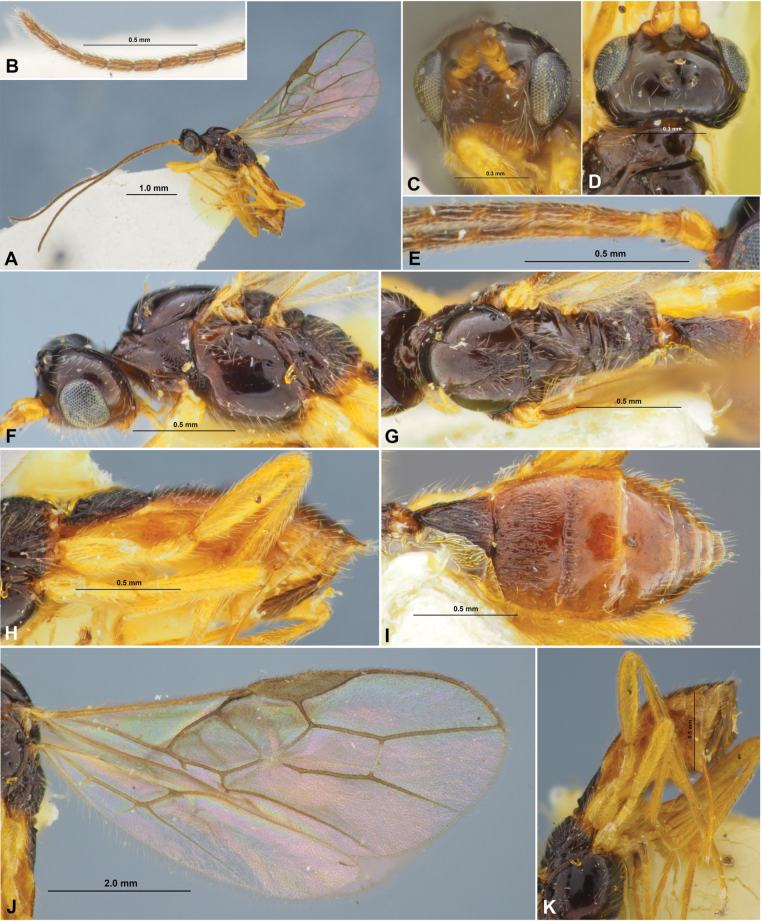
Xenarcha (Xenarcha) pacifica (Belokobylskij), comb. nov., female, holotype **A** habitus, lateral view **B** apical segments of antenna **C** head, front view **D** head and anterior part of mesosoma, dorsal view **E** basal segments of antenna **F** head and mesosoma, lateral view **G** mesosoma and first metasomal tergite, dorsal view **H** metasoma and ovipositor, lateral view **I** metasoma, dorsal view **J** wings **K** metasoma and hind leg, lateral view.

This new subspecies is very similar to Xenarcha (Xenarcha) pacifica ssp. pacifica (Belokobylskij, 1998) (Fig. 8), but differs from later by having the second metasomal tergite striate-rugulose only in anterior 0.3–0.4 (entirely or basically striate-rugulose in X.pacificassp.pacifica).

##### Etymology.

Named from the combination of the Latin words “brevis” (= short) and “sculpta” (= sculptural) because its second tergite of metasoma is sculptured only in the basal 1/2.

##### Distribution.

Korean peninsula.

### ﻿Key to the genera, subgenera, and species of the subfamily Exothecinae of Korean peninsula

**Table:** 

1	Pronotum dorsally without deep round smooth pit (pronope), sometimes only with more or less distinct sculptured transverse groove (Figs 1C, 3C) (Genus *Colastes* Haliday, 1833)	**2**
–	Pronotum dorsally with distinct and deep round smooth pit (pronope) (Figs 5C, 7C, 8D)	**9**
2	Medial lobe of mesoscutum entirely and usually densely setose (Fig. 9A). Body length 2.1–3.3 mm	**C. (Colastes) pubicornis (Thomson, 1892)**
–	Medial lobe of mesoscutum mainly glabrous, setae present only along notauli and in its medio-posterior area (Fig. 9B)	**3**
3	Second tergite of metasoma entirely, third tergite mainly, often fourth tergite in basal 0.3–0.5, and rarely fifth tergite basally striate-rugose (Figs 9C, D). – Notauli complete. Pterostigma yellow or pale brown	**4**
–	Usually only second tergite entirely or at least basally sculptured, but sometimes entirely smooth, following tergites smooth, rarely also only third tergite sculptured in basal 0.2–0.3 (Figs 2C, 9E, F, I)	**5**
4	First metasomal tergite 0.9–1.0× as long as its maximum posterior width. Metasoma posterior to second tergite (pale) reddish brown (Fig. 9C). Transverse diameter of eye 1.4–1.6× longer than temple (dorsal view). First flagellar segment of antenna 1.0–1.1× as long as second segment. Body length 2.7–3.2 mm	**C. (Colastes) sylvicola Belokobylskij, 1998**
–	First metasomal tergite 1.1–1.2× longer than its maximum posterior width. Metasoma entirely dark brown to black (Fig. 9D). Transverse diameter of eye 1.8–2.0× longer than temple (dorsal view). First flagellar segment of antenna 1.2–1.3× longer than second segment. Body length 3.0–3.4 mm	**C. (Colastes) ussuricus Belokobylskij, 1996**
5	Notauli incomplete, distinct in anterior 0.6, very shallow to partly absent in posterior 0.4 (Fig. 1G). Mesoscutum medio-posteriorly entirely smooth (Fig. 1G). Body length 2.1–2.5 mm	**C. (Colastes) fragiloides sp. nov.**
–	Notauli complete, distinct and reaching posterior margin of mesoscutum (Fig. 3G). Mesoscutum medio-posteriorly striate or striate-rugulose at least medially (Fig. 3G)	**6**
6	First flagellar segment of antenna 7.0–8.0× longer than its apical width; penultimate segment 3.5–4.0× longer than its width (Fig. 9G). First tergite of metasoma of female 1.3–1.5× longer than its posterior width (Fig. 9E). Third abscissa of medial vein (2-M) of fore wing in male not widened. Body length 2.2–3.4 mm	**C. (Colastes) dersu Belokobylskij, 1998**
–	First flagellar segment of antenna 3.5–6.0× longer than its apical width; penultimate segment 2.3–3.0× longer than its width (Fig. 9H). First tergite of metasoma of female 1.0–1.1× (rarely 1.20–1.25×) longer than its posterior width (Fig. 9F). Third abscissa of medial vein (2-M) of fore wing in male sometimes widened	**7**
7	Third metasomal tergite rugose-reticulate in its basal 0.3–0.4 (Fig. 4B). Metasoma yellowish brown or yellow in posterior 0.6 (Fig. 4B). First tergite of metasoma of female 1.25× its posterior width. Tibia of hind leg in distal 1/2 and hind tarsus distinctly infuscate (Fig. 3H). Body length 2.8 mm	**C. (Colastes) semiflavus sp. nov.**
–	Third metasomal tergite usually smooth, very rarely anteriorly rugose at short area (Figs 9F, I). Metasoma in posterior 0.6–0.7 reddish brown or dark reddish brown (Figs 9F, I). First tergite of metasoma of female 1.00–1.15× as long as its posterior width (Figs 9F, I). Tibia and tarsus of hind leg yellow	**8**
8	Second metasomal tergite entirely smooth (Fig. 9F). Second metasomal suture very fine (Fig. 9F). Third abscissa of medial vein (2-M) of fore wing in male distinctly widened. Recurrent vein (m-cu) of fore wing subinterstitial or weakly antefurcal to first radiomedial vein (2-SR). Body length 2.5–4.0 mm	**C. (Colastes) braconius Haliday, 1833**
–	Second metasomal tergite entirely or mostly striate with reticulation (Fig. 9I). Second metasomal suture distinct (Fig. 9I). Third abscissa of medial vein (2-M) of fore wing in male not widened. Recurrent vein (m-cu) of fore wing distinctly antefurcal to first radiomedial vein (2-SR). Body length 2.4–3.3 mm	**C. (Colastes) interdictus Belokobylskij, 1998**
9	Second and third metasomal tergites enlarged, forming a subcarapace, posterior segments entirely or almost entirely covered by third tergite (Figs 9J) (Genus *Colastinus* Belokobylskij, 1984). Body length 2.5–3.0 mm	***C.crustatus* Belokobylskij, 1984**
–	Second and third metasomal tergites not enlarged, not forming a subcarapace, posterior segments distinctly protruding beyond third tergite (Figs 9K, L) (Genus *Xenarcha* Foerster, 1863)	**10**
10	Fifth metasomal tergite enlarged and usually completely covering posterior segments (Fig. 9K). First to fifth tergites entirely or almost entirely sculptured (Fig. 9K) (SubgenusPseudophanomeris Belokobylskij, 1984)	**11**
–	Fifth metasomal tergite not enlarged and never covered posterior segments (Figs 9L, 10C–F, K, P, Q). Posterior tergites (fourth and fifth) rarely and only partly (basally) sculptured, often entirely smooth (Figs 9L, 10C–F, K, P, Q)	**12**
11	Medial lobe of mesoscutum entirely covered by dense setae (Fig. 9M). Mesoscutum short, 0.8× as long as its maximum width (Fig. 9M). Transverse diameter of eye ~ 2.0× longer than temple (dorsal view) (Fig. 9O). Face narrow, 1.1–1.2× wider than height of face with clypeus. Body length 2.6–3.0 mm	**X. (P.) pilosa (Belokobylskij, 1984)**
–	Medial lobe of mesoscutum mostly glabrous (Fig. 9N). Mesoscutum long, approx. as long as its maximum width (Fig. 9N). Transverse diameter of eye 1.3–1.5× longer than temple (dorsal view) (Fig. 9P). Face wide, 1.4–1.5× wider than height of face with clypeus. Body length 2.7–3.2 mm	**X. (P.) insularis (Belokobylskij, 1984)**
12	Notauli distinct in anterior 1/2 of mesoscutum, absent in its posterior 1/2; mesoscutum smooth medio-posteriorly (Fig. 10A) (SubgenusShawiana van Achterberg, 1983)	**13**
–	Notauli complete and entirely distinct on mesoscutum; mesoscutum striate or striate-rugose medio-posteriorly (Figs 5G, 7G, 10B) (SubgenusXenarcha s. str.)	**19**
13	Second metasomal tergite entirely smooth (Figs 10C, D)	**14**
–	Second metasomal tergite at least partly (Fig. 10K) or entirely sculptured (Figs 10E, F)	**15**
14	Ovipositor long, its sheath 0.8–1.1× as long as hind tibia, 2.3–2.7× longer than first metasomal tergite (Fig. 10C). Hind tibia distally and hind tarsus entirely yellow or greyish-yellow (Fig. 10G). Body length 2.4–4.5 mm	**X. (Sh.) foveolator (Thomson, 1892)**
–	Ovipositor short, its sheath 0.4–0.5× as long as hind tibia, approx. as long as first metasomal tergite (Fig. 10D). Hind tibia distally and hind tarsus almost entirely distinctly infuscate (Fig. 10H). Body length 2.2–3.5 mm	**X. (Sh.) laevis (Thomson, 1892)**
15	Medial lobe of mesoscutum entirely setose (Fig. 10I). First metasomal tergite narrow, its length 1.2× posterior width (Fig. 10J). Body length 2.4–2.9 mm	**X. (Sh.) rupicola (Belokobylskij, 1998)**
–	Medial lobe of mesoscutum mostly glabrous, setae present only along notauli and in medioposterior area. First metasomal tergite wider, its length usually not larger than posterior width	**16**
16	Basal flagellar segments of antenna longer; first flagellar segment 2.8–4.0× longer than maximum width. – Frons entirely smooth. Second tergite entirely and third at least basally striate with reticulation. Second metasomal tergite 0.6–0.7× as long as its anterior width. Body length 4.1 mm	**X. (Sh.) attonita Papp, 1987**
–	Basal flagellar segments of antenna shorter; first flagellar segment 2.0–2.5× longer than its maximum width (Figs 10L, M)	**17**
17	Second metasomal tergite only partly sculptured, its sides and posterior margin (sometimes entirely in posterior 1/2) smooth (Fig. 10K). Antenna reddish brown basally (Fig. 10L). Body length 2.3–3.3 mm	**X. (Sh.) nupta (Papp, 1983)**
–	Second metasomal tergite entirely sculptured (Fig. 10F). Antenna usually pale reddish brown basally (Fig. 10M)	**18**
18	Third metasomal tergite rugose-striate in anterior 0.2–0.5; fourth tergite entirely smooth (Fig. 9L). Body length 3.0–5.0 mm	**X. (Sh.) catenator (Haliday, 1836)**
–	Third metasomal tergite mainly or entirely rugose-striate; fourth tergite sculptured basally (Fig. 10F). Body length 2.5–3.3 mm	**X. (Sh.) orientalis (Belokobylskij, 1998)**
19	Ovipositor long, its sheath 1.0–1.5× as long as hind tibia, 2.1–2.7× longer than first metasomal tergite (Fig. 10O). Precoxal sulcus weakly present (Fig. 10N). – Second tergite of metasoma striate only in antero-medial part (Fig. 10P). Body length 2.7–3.4 mm	**X. (X.) effecta (Papp, 1972)**
–	Ovipositor short, its sheath 0.5–0.7× as long as hind tibia, approx. as long as first metasomal tergite (Fig. 10Q). Precoxal sulcus always absent	**20**
20	Third metasomal tergite entirely striate, with more or less distinct oblique antero-lateral furrows (Fig. 10Q). Temple short, transverse diameter of eye 2.0–2.5× longer than temple (dorsal view) (Fig. 10R). Body length 1.6–2.7 mm	**X. (X.) ivani (Belokobylskij, 1986)**
–	Third metasomal tergite usually entirely smooth, rarely with short basal striae, without oblique antero-lateral furrows (Figs 6B, 7I). Temple long, transverse diameter of eye 1.3–1.7× longer than temple (dorsal view) (Figs 5C, 7C)	**21**
21	Temple (dorsal view) strongly curvedly narrowed behind eye (Fig. 5C). Penultimate segment of antenna 2.7–2.8× longer than its width (Fig. 5D). Pronotum dorsally with wide pronope and high, medially curved pronotal carina (Fig. 5C). First metasomal tergite 1.2–1.3× longer than its anterior width (Fig. 6B). Body length 3.1–4.1 mm	**X. (X.) pacificoformis sp. nov.**
–	Temple (dorsal view) not strongly and convex-curvedly narrowed behind eye (Fig. 7C). Penultimate segment of antenna 2.0–2.3× longer than its width (Fig. 7D). Pronotum dorsally with narrow pronope and without pronotal carina (Fig. 7C). First metasomal tergite 1.0–1.1× longer than its anterior width (Fig. 7K). Body length 3.0–3.5 mm	**X. (X.) pacifica (Blkb) ssp. brevisculpta ssp. nov.**

**Figure 9.**
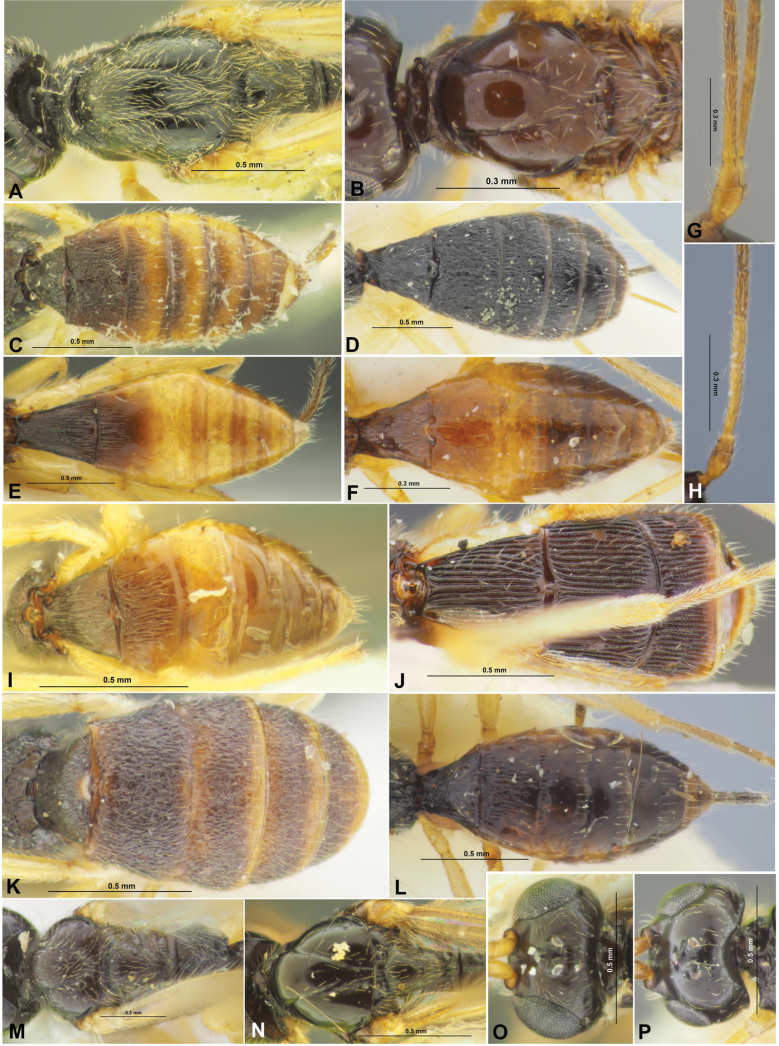
Morphological details of Colastes (Colastes) pubicornis (**A**), C. (C.) braconius (**B, F, H**), C. (C.) sylvicola (**C**), C. (C.) ussuricus (**D**), C. (C.) dersu (**E, G**), C. (C.) interdictus (**I**), *Colastinuscrustatus* (**J**), Xenarcha (Pseudophanomeris) pilosa (**K, M, O**), X. (Shawiana) catenator (**L**), X. (Ps.) insularis (**N, P**). **A, B, M, N** Mesoscutum and scutellum, dorsal view **C–F, I–L** metasoma, dorsal view **G, H** basal segments of antenna **O, P** head, dorsal view.

**Figure 10.**
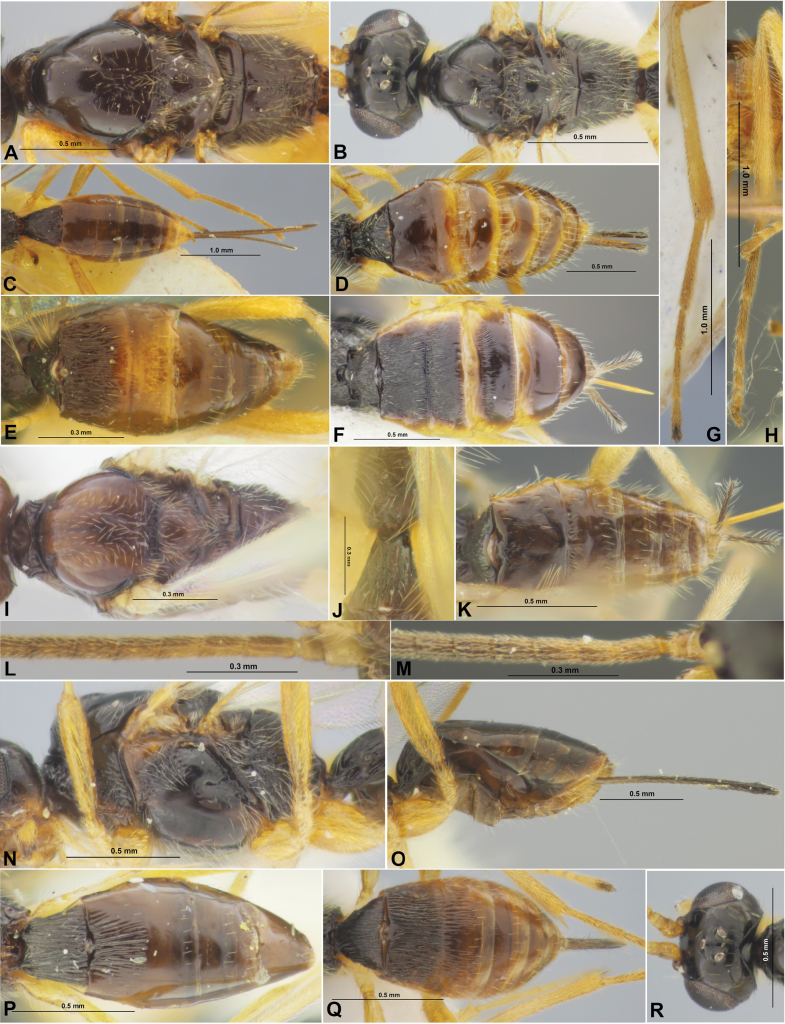
Morphological details of Xenarcha (Shawiana) foveolator (**A, C, G**), X. (Xenarcha) ivani (**B, Q, R**), X. (Sh.) laevis (**D, H**), X. (Sh.) rupicola (**E, I, J**), X. (Sh.) orientalis (**F**), X. (Sh.) nupta (**K, L**), X. (Sh.) catenator (**M**), X. (X.) effecta (**N, O, P**). **A** Mesosoma, dorsal view **B** head and mesosoma, dorsal view **C–F, K, P, Q** metasoma and ovipositor, dorsal view **G, H** tibia and tarsus of hind leg **I** mesoscutum and scutellum, dorsal view **J** propodeum and first tergite of metasoma, dorsal view **L, M** basal segments of antenna **N** mesosoma, lateral view **O** metasoma and ovipositor, lateral view **R** head, dorsal view.

## ﻿Discussion

The generic composition of the subfamily Exothecinae s. str. has been changed several times during its modern history (van Achterberg 1995). The tribe Exothecini (sensu Belokobylskij 1993) included the following genera: *Colastes* (with subgenera *Pseudophanomeris* and *Shawiana*), *Colastinus*, *Vietcolastes* and *Xenarcha*. On the other hand, *Shawiana* was treated as a separate valid genus for a long time (van Achterberg 1983; Whitfield and Wharton 1997; Yu et al. 2016), but Belokobylskij (1998) included this name (as well as *Xenarcha*) as a subgenus of the genus *Colastes* sensu lato. However, the eastern Asian genera *Colastinus* Belokobylskij, 1984, *Orientocolastes* Belokobylskij, 1999 and *Vietcolastes* Belokobylskij, 1992 described later (Belokobylskij 1984, 1992, 1999) maintained their status without changing until now.

The first molecular-phylogenetic study using the 28S and COI genes for more than single Exothecinae supraspecies taxa (genera or subgenera) (*Colastes*, *Pseudophanomeris*, *Shawiana*, and *Xenarcha*) was provided by Zaldivar-Riverón et al. (2006). This study revealed the separate position of *Colastes* from the other three taxa which were united in a separate clade. Overall, the member of Exothecinae was showed a distinct phylogenetic relation with the subfamilies Alysiinae and Opiinae. Similar results were obtained in other analyses of the cyclostome braconids however with using only single gene 28S (D2-D3) (Ranjith et al. 2017), but the two studied here *Colastes* species (*C.braconius* Haliday and *C.incertus* (Wesmael, 1838)) were separated in different but related clades. Finally, a large mitochondrial phylogenomic study of the Braconidae subfamilies (Jasso-Martínez et al. 2022) have been also showed a distinct relation between the taxa *Pseudophanomeris*, *Xenarcha*, and *Shawiana* (with very high bootstrap values), but they were nested separately in the same clade together with *Vietcolastes* and *Colastinus*.

**Figure 11.**
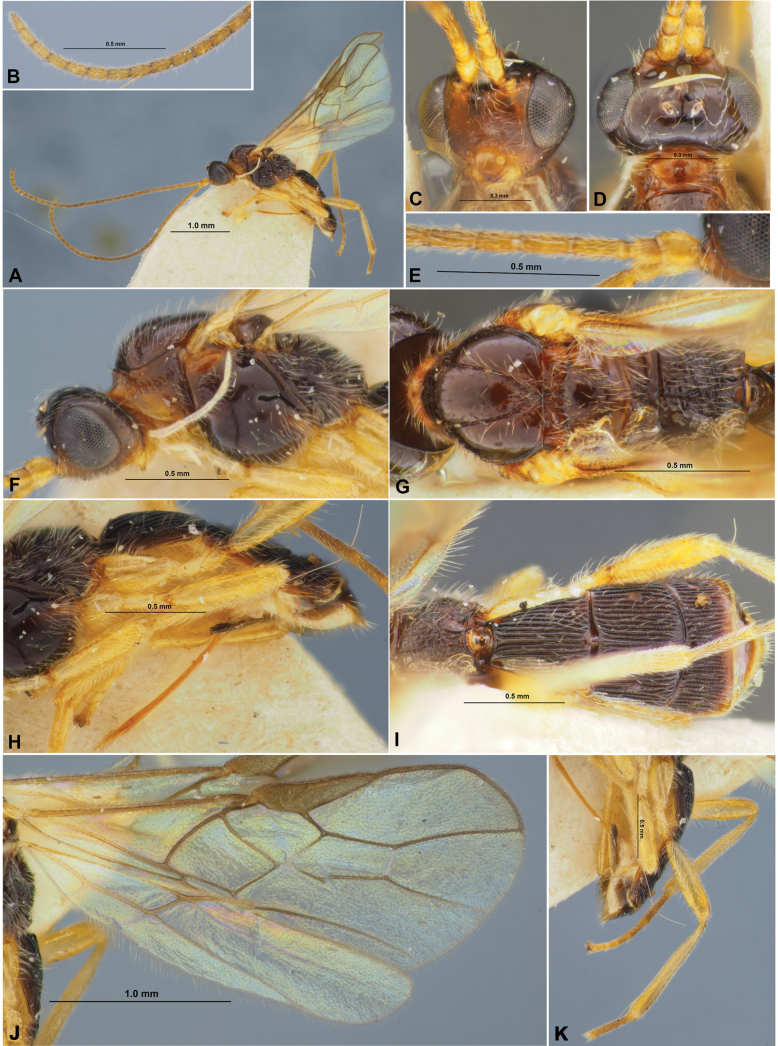
*Colastinuscrustatus* Belokobylskij, female, holotype **A** habitus, lateral view **B** apical segments of antenna **C** head, front view **D** head and anterior part of mesosoma, dorsal view **E** basal segments of antenna **F** head and mesosoma, lateral view **G** mesosoma, dorsal view **H** metasoma and ovipositor, lateral view **I** propodeum and metasoma, dorsal view **J** wings **K** metasoma and hind leg, lateral view.

**Figure 12.**
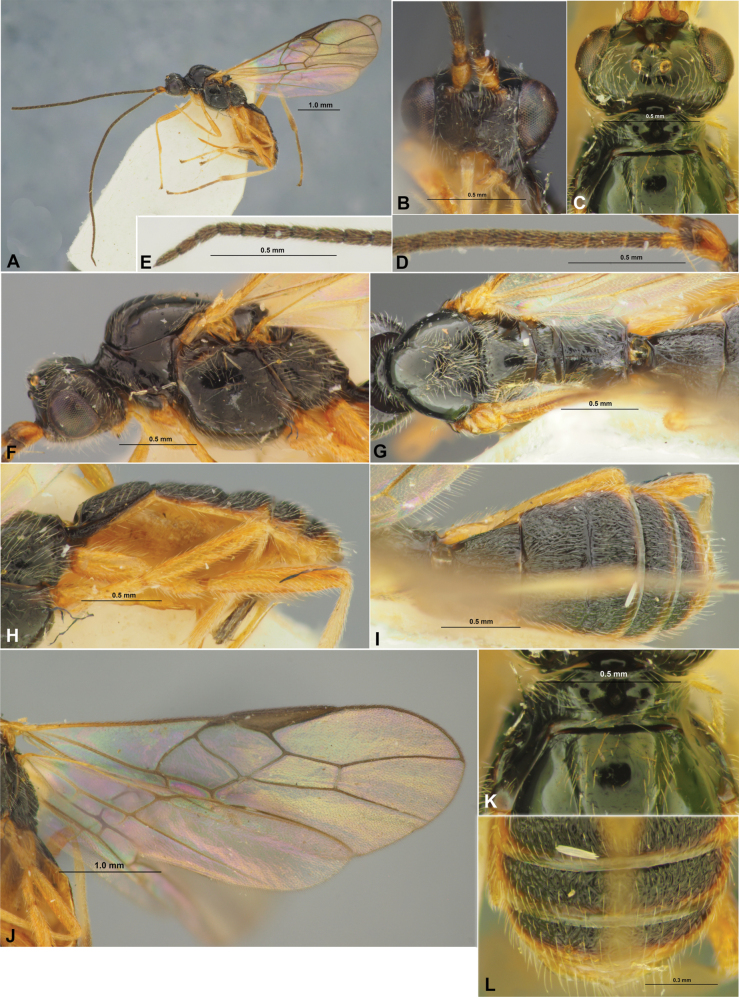
Xenarcha (Pseudophanomeris) unicolor (Belokobylskij), female, holotype **A** habitus, lateral view **B** head, front view **C** head and anterior part of mesosoma, dorsal view **D** basal segments of antenna **E** apical segments of antenna **F** head and mesosoma, lateral view **G** mesosoma and first metasomal tergite, dorsal view **H** propodeum, metasoma and ovipositor, lateral view **I** propodeum and metasoma, dorsal view **J** wings **K** anterior part of mesosoma, dorsal view **L** posterior part of metasoma, dorsal view.

**Figure 13.**
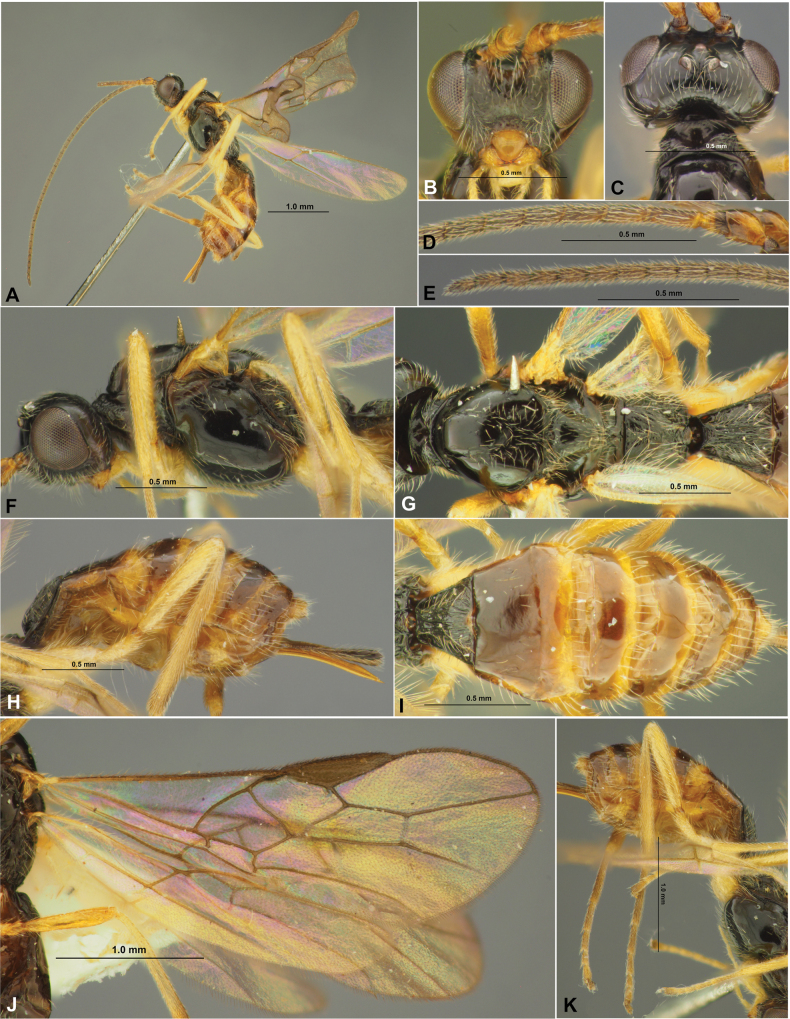
Xenarcha (Shawiana) laevis (Thomson), female **A** habitus, lateral view **B** head, front view **C** head and anterior part of mesosoma, dorsal view **D** basal segments of antenna **E** apical segments of antenna **F** head and mesosoma, lateral view **G** mesosoma and first metasomal tergite, dorsal view **H** metasoma and ovipositor, lateral view **I** metasoma, dorsal view **J** wings **K** metasoma and hind leg, lateral view.

**Figure 14.**
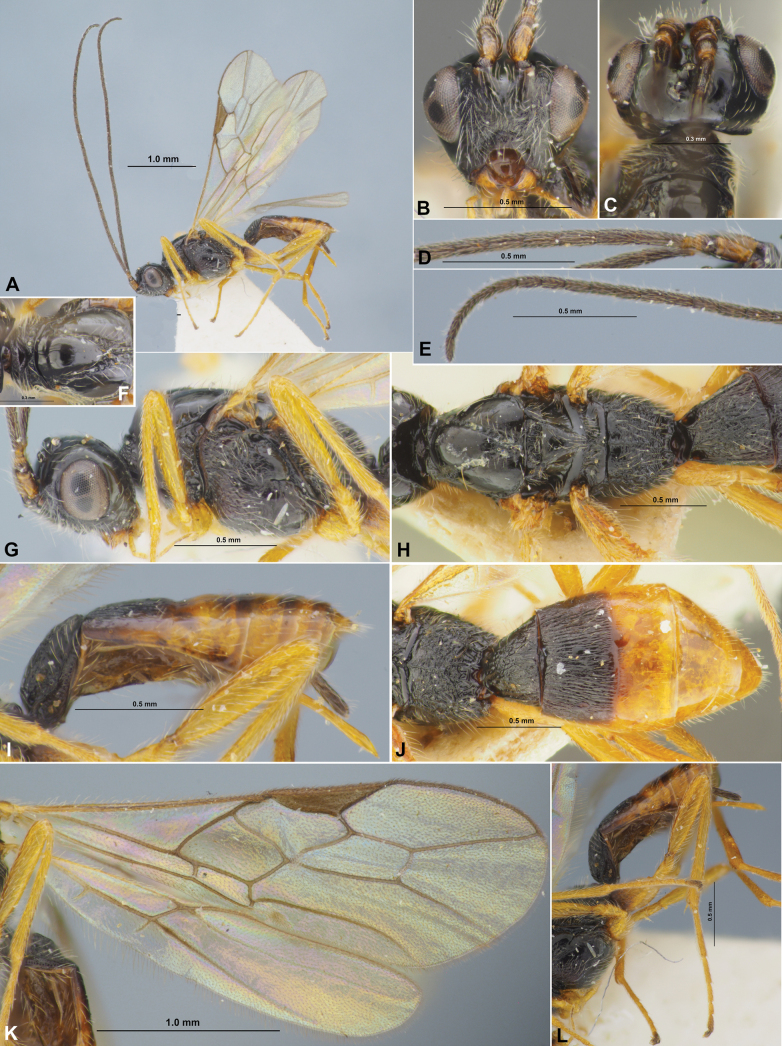
Xenarcha (Xenarcha) lustrator (Haliday), female **A** habitus, lateral view **B** head, front view **C** head and anterior part of mesosoma, dorsal view **D** basal segments of antenna **E** apical segments of antenna **F** anterior half of mesosoma **G** head and mesosoma, lateral view **H** mesosoma and first metasomal tergite, dorsal view **I** metasoma and ovipositor, lateral view **J** propodeum and metasoma, dorsal view **K** wings **L** metasoma and hind leg, lateral view.

**Figure 15.**
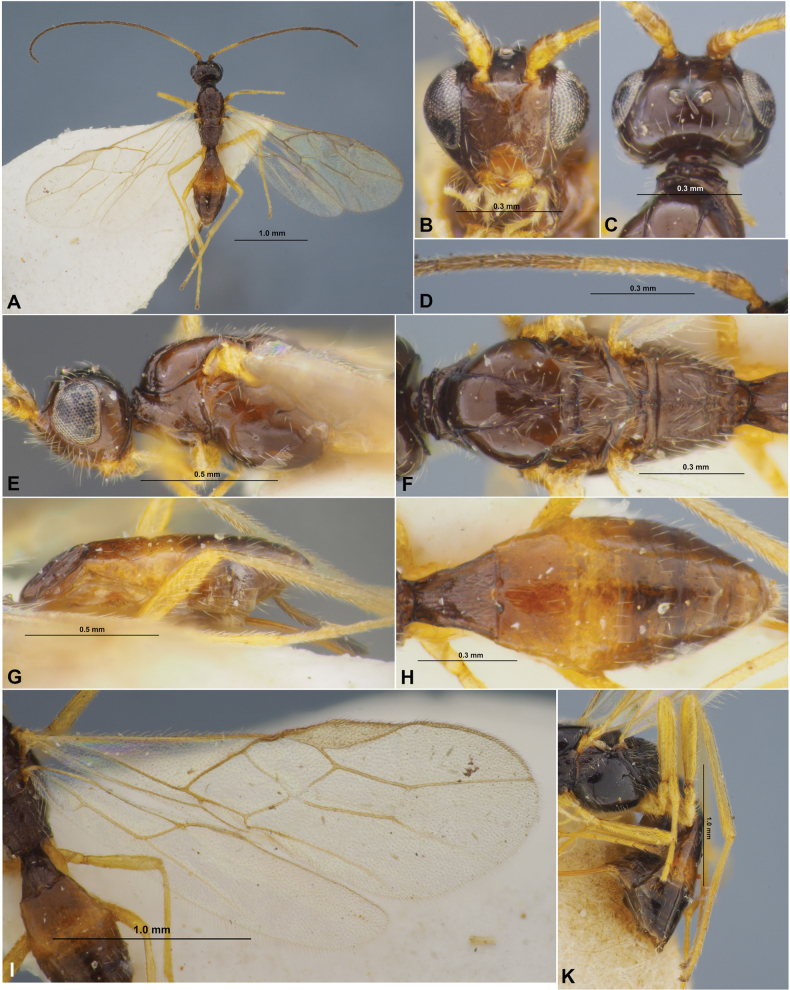
Colastes (Colastes) braconius Haliday, female **A** habitus, dorsal view **B** head, front view **C** head and anterior part of mesosoma, dorsal view **D** basal segments of antenna **E** head and mesosoma, lateral view **F** mesosoma, dorsal view **G** metasoma and ovipositor, lateral view **H** metasoma, dorsal view **I** wings **K** metasoma and hind leg, lateral view.

**Figure 16.**
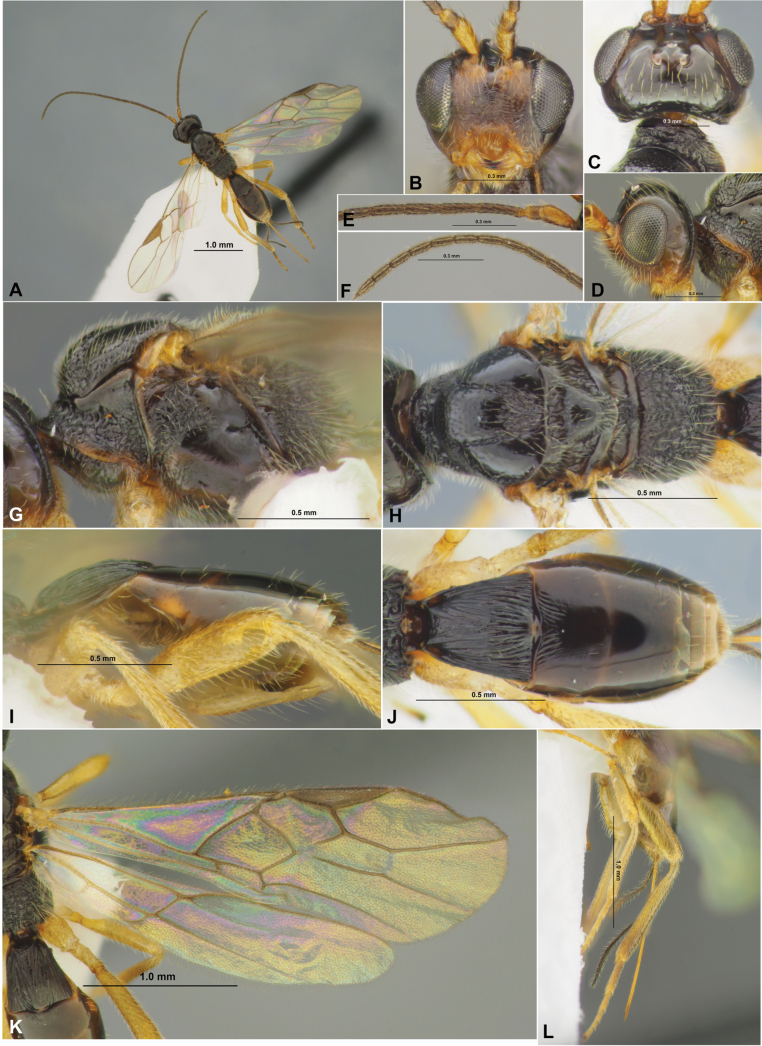
Colastes (Fungivenator) fritzeni van Achterberg & Shaw, female **A** habitus, dorsal view **B** head, front view **C** head and anterior part of mesosoma, dorsal view **D** head and anterior part of mesosoma, lateral view **E** basal segments of antenna **F** apical segments of antenna **G** mesosoma, lateral view **H** mesosoma, dorsal view **I** metasoma, lateral view **J** metasoma, dorsal view **K** wings **L** hind leg, lateral view.

**Figure 17.**
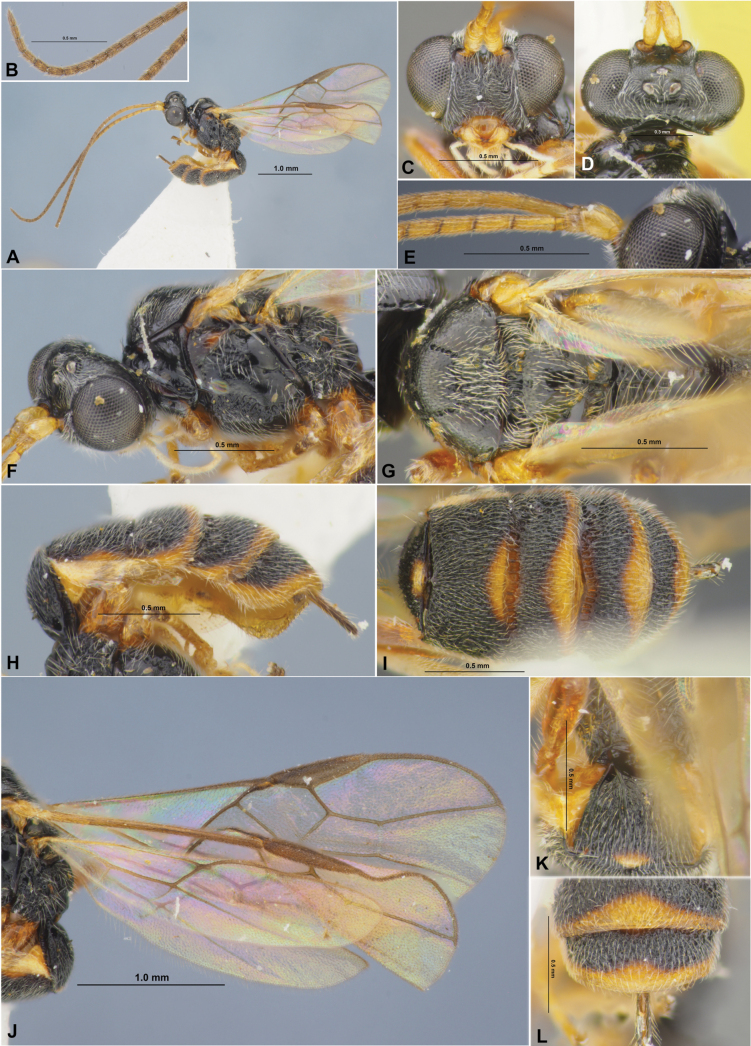
*Orientocolastesio* Belokobylskij, female **A** habitus, lateral view **B** apical segments of antenna **C** head, front view **D** head and anterior part of mesosoma, dorsal view **E** basal segments of antenna **F** head and mesosoma, lateral view **G** mesosoma, dorsal view **H** metasoma and ovipositor, lateral view **I** metasoma, dorsal view **J** wings **K** propodeum and first metasomal tergite, dorsal view **L** posterior part of metasoma, dorsal view.

**Figure 18.**
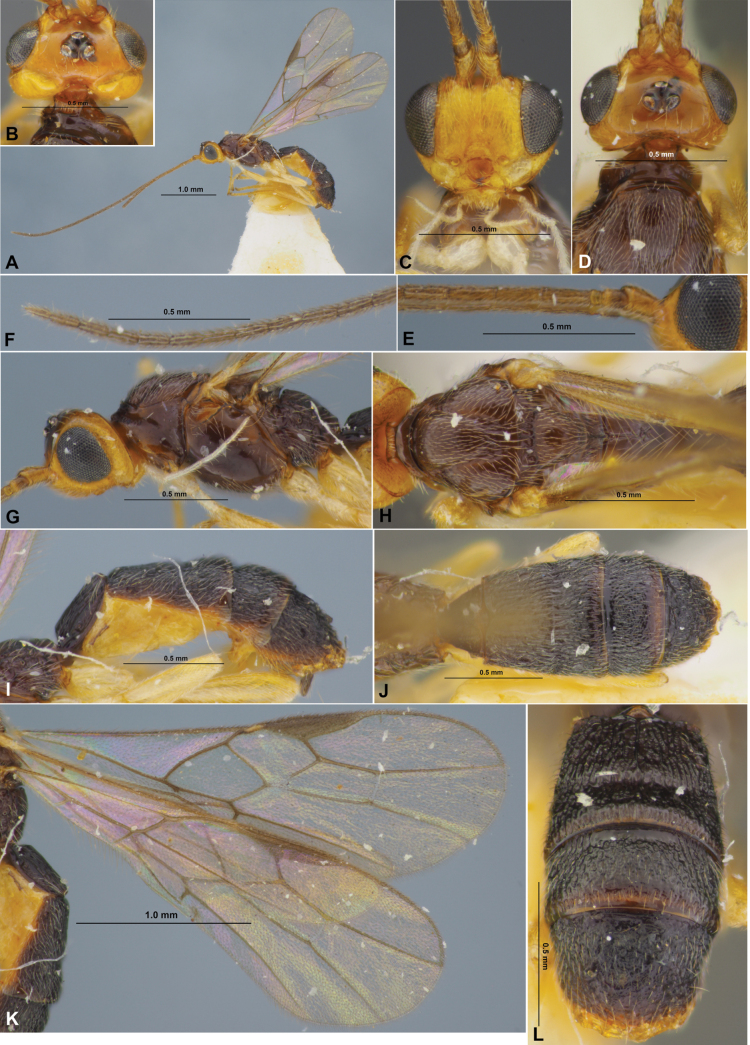
*Vietcolastesrhaconotus* Belokobylskij, female, holotype (**A, C–L**), and male, paratype (**B**) **A** habitus, lateral view **B** head, dorsal view **C** head, front view **D** head and anterior part of mesosoma, dorsal view **E** basal segments of antenna **F** apical segments of antenna **G** head and mesosoma, lateral view **H** mesosoma, dorsal view **I** propodeum, metasoma and ovipositor, lateral view **J** propodeum and metasoma, dorsal view **K** wings **L** posterior part of metasoma, dorsal view.

Our morphological study of Exothecinae taxa showed that the genera of this subfamily are divided into two morphological groups based on pronotal structure: dorsally with distinct, wide, deep and mainly smooth round pit (pronope) or without pronope (however rarely with a distinct transverse sculptured groove). The genera *Colastinus* (Fig. 11) and *Xenarcha* [including subgenera *Pseudophanomeris* (Fig. 12), *Shawiana* (Fig. 13) and *Xenarcha* s. str. (Fig. 14)] belong to the first group (with pronope). The second morphological group (without pronope) includes *Colastes* [with subgenera *Colastes* s. str. (Fig. 15), *Discolastes* Belokobylskij, 2000 and *Fungivenator* van Achterberg & Shaw, 2008 (Fig. 16)], *Orientocolastes* (Fig. 17), and *Vietcolastes* (Fig. 18). A key for determination of Exothecinae genera and subgenera is provided below.

### ﻿Key to the world Exothecinae genera and subgenera

**Table:** 

1	Pronotum dorsally with distinct, deep and rather wide round and usually smooth pit (pronope) (Figs 11D, 12C, 13C, 14C)	**2**
–	Pronotum dorsally without deep round pit (pronope), sometimes only with transverse sculptured groove (Figs 15C, 16C, 17D, 18D)	**5**
2	Second and third metasomal tergites forming a carapace, covering or almost covering the following segments. Third tergite with flange on posterior margin (Fig. 11I). Oriental, Palaearctic regions	***Colastinus* Belokobylskij, 1984**
–	Second and third metasomal tergites not forming a carapace, following segments (at least till fifth one) distinctly protruding behind third one. Third tergite without flange on posterior margin (Figs 12I, 13I, 14J). Afrotropical, Oriental, Palaearctic regions (*Xenarcha* Foerster, 1863)	**3**
3	Fifth metasomal tergite enlarged and usually covered posterior segments (Fig. 12I). First to fifth tergites entirely sculptured (Fig. 12I). Afrotropical, Oriental, Palaearctic regions	***Xenarcha*** (***Pseudophanomeris* Belokobylskij, 1984**)
–	Fifth metasomal tergite not enlarged and never covering posterior segments (Figs 13I, 14J). Posterior tergites (fourth and fifth) rarely and only partly (anteriorly) sculptured, often entirely or mostly smooth (Figs 13I, 14J)	**4**
4	Notauli incomplete, absent in posterior 0.3–0.5 of mesoscutum; mesoscutum predominantly smooth in medioposterior part (Fig. 13G). Radial vein (r) of fore wing usually arising from basal 0.3–0.4 of pterostigma (Fig. 13J). Nearctic, Oriental, Palaearctic regions	***Xenarcha*** (***Shawiana* van Achterberg, 1983**)
–	Notauli complete, reaching postrior margin of mesoscutum; mesoscutum always sculptured in its medioposterior part (Fig. 14H). Radial vein (r) of fore wing usually arising from basal 0.4–0.5 of pterostigma (Fig. 14K). Nearctic, Oriental, Palaearctic regions	***Xenarcha* (*Xenarcha* s. str.**)
5	Occipital carina present dorsally (Fig. 17D). Frons with sculptured longitudinal furrow. Precoxal sulcus present and distinct (Fig. 17F). Prepectal carina present laterally (Fig. 17F). Oriental region	***Orientocolastes* Belokobylskij, 1999**
–	Occipital carina widely interrupted dorsally (Figs 15C, 16C, 18D). Frons without sculptured longitudinal furrow (Figs 15C, 16C, 18D). Precoxal sulcus usually absent (Figs 15E, 16G, 18G), if rarely present then very shallow. Prepectal carina completely absent (Figs 15E, 16G, 18G)	**6**
6	Fifth metasomal tergite enlarged and covering posterior segments (Figs 18J, L). First to fifth tergite entirely sculptured (Fig. 18J). In male, occipital carinae absent; temple with distinct lateral vertical occipital lumps (Fig. 18B). Oriental region	***Vietcolastes* Belokobylskij, 1992**
–	Fifth metasomal tergite not enlarged and never covering posterior segments (Figs 15H, 16J). Posterior tergites (fourth and fifth) rarely only partly (basally) sculptured, often entirely or mostly smooth (Figs 15H, 16J). In male, occipital carinae always present laterally; temple without lateral vertical occipital lumps. Nearctic, Neotropical, Oriental, Palaearctic regions (*Colastes* Haliday, 1833)	**7**
7	Notauli complete and widely separated in posterior margin of mesoscutum. Propodeum smooth, with areola delineated by distinct carinae. Precoxal sulcus present, but fine, with distinct longitudinal carina on its posterior margin. Oriental region	***Colastes* (*Discolastes* Belokobylskij, 2000**)
–	Notauli complete or incomplete, if complete, than always fused near posterior margin of mesoscutum (Figs 15F, 16H). Propodeum usually mostly sculptured and often without areola, if sometimes areola present, than delineated (often only partly) by weak carinae. Precoxal sulcus predominantly absent, longitudinal carina on lower margin of mesopleuron absent	**8**
8	Ventral rim of clypeus thick, situated at same level as that of face and apex of mandible (lateral view) (Fig. 15E). Scutellum smooth medio-posteriorly (Fig. 15F). Parasitoid of leaf-mining larvae and of larvae in leaf-galls. Australasian, Nearctic, Neotropical, Oriental, Palaearctic regions	***Colastes*** (***Colastes* s. str.**)
–	Ventral rim of clypeus thin, protruding beyond level of face and apex of mandible (lateral view) (Fig. 16D). Scutellum often rugulose medio-posteriorly (Fig. 16H). Parasitoids of larvae in bracket fungi. – Notauli posteriorly meeting in rugose-striate medio-posterior area (Fig. 16H). Nearctic, Palaearctic regions	***Colastes*** (***Fungivenator* van Achterberg & Shaw, 2008**)

## Supplementary Material

XML Treatment for Colastes

XML Treatment for Colastes (Colastes) braconius

XML Treatment for Colastes (Colastes) dersu

XML Treatment for Colastes (Colastes) fragiloides

XML Treatment for Colastes (Colastes) interdictus

XML Treatment for Colastes (Colastes) pubicornis

XML Treatment for Colastes (Colastes) semiflavus

XML Treatment for Colastes (Colastes) sylvicola

XML Treatment for Colastes (Colastes) ussuricus

XML Treatment for Colastinus

XML Treatment for Colastinus crustatus

XML Treatment for Xenarcha

XML Treatment for Subgenus Pseudophanomeris

XML Treatment for Xenarcha (Pseudophanomeris) insularis

XML Treatment for Xenarcha (Pseudophanomeris) pilosa

XML Treatment for Subgenus Shawiana

XML Treatment for Xenarcha (Shawiana) attonita

XML Treatment for Xenarcha (Shawiana) catenator

XML Treatment for Xenarcha (Shawiana) foveolator

XML Treatment for Xenarcha (Shawiana) laevis

XML Treatment for Xenarcha (Shawiana) nupta

XML Treatment for Xenarcha (Shawiana) orientalis

XML Treatment for Xenarcha (Shawiana) rupicola

XML Treatment for Xenarcha (Xenarcha) effecta

XML Treatment for Xenarcha (Xenarcha) ivani

XML Treatment for Xenarcha (Xenarcha) pacificoformis

XML Treatment for Xenarcha (Xenarcha) pacifica(Belokobylskij, 1998)ssp.brevisculpta

## ﻿Additional information

### Author ORCIDs

Sergey A. Belokobylskij https://orcid.org/0000-0002-3646-3459

Deokseo Ku https://orcid.org/0000-0002-6274-6479

### Data availability

All of the data that support the findings of this study are available in the main text.

## References

[B1] Belokobylskij SA (1984) On the division of the tribe Exothecini s.l. (Hymenoptera, Braco­nidae) in two with the description of a new genus and subgenus. Zoologicheskiy Zhurnal 63: 1019–1026. [In Russian]

[B2] Belokobylskij SA (1986) A new braconid species of the supertribe Exothecidii (Hymenoptera, Braconidae) from south of the USSR Far East. In: Lehr PA (Ed.) Systematika i Ekologiya Nasekomych Dalinego Vostoka.[Systematics and ecology of the insects from the Far East.] Vladivostok, 58–69. [In Russian]

[B3] Belokobylskij SA (1990) A contribution to the braconid fauna (Hymenoptera) of the Far East. Vestnik Zoologii 1990(6): 32–39. [In Russian]

[B4] Belokobylskij SA (1992) Contribution to the fauna of the Indo-Malayan braconid wasps of the tribe Exothecini, Pambolini and Pentatermini (Hymenoptera, Braconidae). Proceedings of the Zoological Institute 245: 125–173. [In Russian]

[B5] Belokobylskij SA (1993) On the classification and phylogeny of the braconid wasps subfamilies Doryctinae and Exothecinae (Hymenoptera, Braconidae). Part 1. On the classification, 2. Entomologicheskoe Obozrenie 72(1): 143–164. [In Russian]

[B6] Belokobylskij SA (1994) A review of parasitic wasps of the subfamilies Doryctinae and Exothecinae (Hymenoptera, Braconidae) of the Far East, eastern Siberia and neighbouring territories. In: Kotenko AG (Ed.) Hymenopteran insects of Siberia and Far East.Memoirs of the Daurskiy Nature Reserve 3: 5–77. [In Russian]

[B7] Belokobylskij SA (1996) Nine new species of Braconidae (Hymenoptera) from the Russian Far East. Journal of Natural History 30(11): 1661–1681. 10.1080/00222939600770981

[B8] Belokobylskij SA (1998) Subfam. Exothecinae. In: Lehr PA (Ed.) Key to insects of the Russian Far East.Vol. 4. Neuropteroidea, Mecoptera, Hymenoptera. Pt 3. Dal’nauka, Vladivostok, 111–159. [In Russian]

[B9] Belokobylskij SA (1999) New genera of the subfamilies Rhyssalinae, Exothecinae and Gnamptodontinae from the Old World (Hymenoptera: Braconidae). Zoosystematica Rossica 8(1): 155–169.

[B10] Belokobylskij SA Maetô K (2009) Doryctinae (Hymenoptera, Braconidae) of Japan. Fauna mundi. Vol. 1. Warszawska Drukarnia Naukowa, Warszawa, 806 pp.

[B11] Belokobylskij SA Tobias VI (1986) Subfam. Doryctinae. In: Medvedev GS (Ed.) Keys to insects of the USSR European part.Vol. 4. Hymenoptera. Pt. 4. Nauka, Leningrad, 21–72. [In Russian]

[B12] Belokobylskij SA Samartsev KG Il’inskaya AS [Eds] (2019) Annotated catalogue of the Hymenoptera of Russia. Volume II. Apocrita: Parasitica. Proceedings of the Zoological Institute Russian Academy of Sciences. Supplement 8. St Petersburg, 594 pp. 10.31610/trudyzin/2019.supl.8.5

[B13] Belokobylskij SA Ku D-S Lee H-R Kwon G-M (2024) Braconidae: Rhyssalinae and Doryctinae. Arthropoda: Insecta: Hymenoptera. Insect Fauna of Korea 13(16): 1–513.

[B14] Haliday AH (1833) An essay on the classification of the parasitic Hymenoptera of Britain, which correspond with the Ichneumones minuti of Linnaeus. Entomological Magazine 1(iii): 259–276, 333–350.

[B15] Haliday AH (1836) Essay on parasitic Hymenoptera. Entomological Magazine 4(ii): 92–106.

[B16] Jasso-Martínez JM Quicke DLJ Belokobylskij SA Santos BF Fernández-Triana JL Kula RR Zaldivar-Riverón A (2022) Mitochondrial phylogenomics and mitogenome organization in the parasitoid wasp family Braconidae (Hymenoptera: Ichneumonoidea). BMC Ecology and Evolution 22: 46. 10.1186/s12862-022-01983-1 PMC9006417 35413835

[B17] Ku DS Belokobylskij SA Cha JY (2001) Hymenoptera (Braconidae). Economic Insects of Korea 16. Insecta Koreana. Supplement 23: 1–283.

[B18] Martínez JJ Diez F (2024) Taxonomic notes on some neglected doryctine wasps (Hymenoptera: Braconidae) from Argentina described by J. Brèthes and E. Blanchard. Zootaxa 5406(1): 190–200. 10.11646/zootaxa.5406.1.11 38480156

[B19] Papp J (1972) *Exothecuseffectus* sp. n., a new braconid species from N.E. China (Hym.). Acta Zoologica Academiae Scientiarum Hungaricae 18: 323–325.

[B20] Papp J (1975) Three new European species of *Colastes* Hal. with taxonomic remarks (Hym., Braconidae, Exothecinae). Acta Zoologica Academiae Scientiarum Hungaricae 21: 411–423.

[B21] Papp J (1983) Braconidae (Hymenoptera) from Mongolia. IX. Acta Zoologica Academiae Scientiarum Hungaricae 29: 441–449.

[B22] Papp J (1987) Braconidae (Hymenoptera) from Korea. VIII. Acta Zoologica Academiae Scientiarum Hungaricae 33: 157–175.

[B23] Papp J (1992) Braconidae (Hymenoptera) from Korea. XIV. Acta Zoologica Academiae Scientiarum Hungaricae 38: 63–73.

[B24] Papp J (2003) Braconidae (Hymenoptera) from Korea, XXI. Species of fifteen subfamilies. Acta Zoologica Academiae Scientiarum Hungaricae 49: 115–152.

[B25] Ranjith AP Belokobylskij SA Quicke DLJ Kittel RN Butcher BA Nasser M (2017) An enigmatic new genus of Hormiinae (Hymenoptera: Braconidae) from South India. Zootaxa 4272(3): 371–385. 10.11646/zootaxa.4272.3.3 28610281

[B26] Shenefelt RD (1975) Braconidae 8. Exothecinae, Rogadinae. Hymenopterorum Catalogus, Nova Editio, Pars 12: 1115–1262.

[B27] Thomson CG (1892) XLIV. Bidrag till Braconidernas kannedom. Opuscula Entomologica 16: 1659–1751.

[B28] van Achterberg C (1983) Revisionary notes on the Palaearctic genera and species of the tribe Exothecini Foerster (Hymenoptera: Braconidae). Zoologische Mededelingen 57(26): 339–355.

[B29] van Achterberg C (1993) Illustrated key to the subfamilies of the Braconidae (Hymenoptera: Ichneumonoidea). Zoologische Verhandelingen 283: 1–189.

[B30] van Achterberg C (1995) Generic revision of the subfamily Betylobraconinae (Hymenoptera: Braconidae) and other groups with modified fore tarsus. Zoologische Verhandelingen 298: 1–242.

[B31] van Achterberg C Shaw MR (2008) A new subgenus of the genus *Colastes* Haliday (Hymenoptera: Braconidae: Exothecinae) for species reared from bracket fungi, with description of two new species from Europe. Journal of Natural History 42(27–28): 1849–1860. 10.1080/00222930802114280

[B32] Wesmael C (1838) Monographie des Braconides de Belgique. 4. Nouveaux Mémoires de l’Academie Royale des Sciences et Belles-lettres de Bruxelles 11: 1–166. 10.3406/marb.1837.2702

[B33] Whitfield JB Wharton RA (1997) Hormiinae. In: Wharton RA Marsh PM Sharkey MJ (Eds) Manual of the New World genera of the family Braconidae (Hymenoptera). International Society of Hymenopterists. Special Publication No.1: 285–301.

[B34] Yu DS van Achterberg C Horstmann K (2016) Taxapad 2016. Ichneumonoidea 2015. Nepean, Ottawa, Ontario. [Database on flash-drive]

[B35] Zaldivar-Riverón A Mori M Quicke DLJ (2006) Systematics of the cyclostome subfamilies of braconid parasitic wasps (Hymenoptera: Ichneumonoidea): a simultaneous molecular and morphological Bayesian approach. Molecular Phylogenetics and Evolution 38(1): 130–145. 10.1016/j.ympev.2005.08.006 16213168

